# Performance Optimization of Electrospun Lithium-Ion Conducting PAN/PEO Solid Polymer Electrolyte

**DOI:** 10.1021/acs.inorgchem.5c03238

**Published:** 2025-09-25

**Authors:** Elisabeth B. Springl, Diganta Sarkar, Marvin Mühlau, Vladimir K. Michaelis, Tom Nilges

**Affiliations:** † School of Natural Sciences (NAT), Department of Chemistry, Technische Universität München, Lichtenbergstraße 4, Garching bei München 85748, Germany; ‡ TUMint.Energy Research GmbH, Lichtenbergstraße 4, Garching bei München 85748, Germany; § Department of Chemistry, 3158 University of Alberta, Edmonton, Alberta T6G 2G2, Canada

## Abstract

Storing energy in rechargeable lithium-ion batteries is essential for a renewable energy supply. Replacing liquid with solid electrolytes in all-solid-state batteries minimizes safety concerns while increasing energy density. This study introduces a solid polymer electrolyte membrane that can be produced in a scalable, one-step process. The polymer blend consisting of the matrix-giving polyacrylonitrile (PAN) and the ion-conducting poly­(ethylene oxide) (PEO) with lithium bis­(trifluoromethanesulfonyl)­imide (LiTFSI) as the conducting salt is electrospun, ensuring mechanical flexibility and low crystallinity. Flexible, free-standing membranes exhibit fiber retention up to 100 °C, enabling a wide thermal application window above PEO’s melting point. Adjusting the plasticizer ratio, humidity, and drying conditions allows fine-tuning of the membrane’s morphology, porosity, and ionic conductivity, reaching 0.1 mS cm^–1^ at 328 K. A slight increase in cell pressure from 0.6 to 2.1 MPa decreases porosity and further increases ionic conductivity without affecting the fiber structure, enabling low-pressure utilization. Moreover, variable-temperature ^7^Li solid-state nuclear magnetic resonance spectroscopy studies of the dry membrane further demonstrated rapid local Li-ion exchange processes with very low activation energies. An electrochemical window between 0 and 4.5 V, and reversible lithium-ion transport, confirmed by galvanostatic cycling, imply the promising application of high-performance electrospun solid polymer electrolytes.

## Graphical abstract

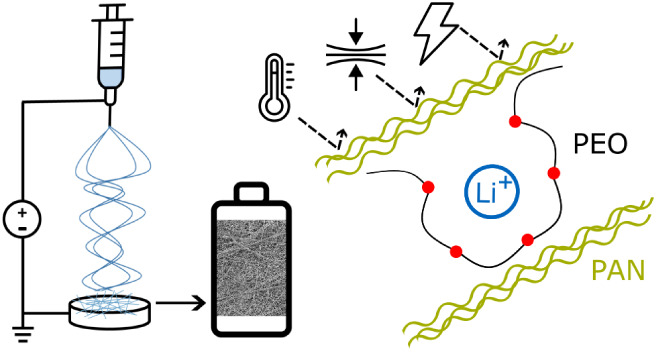

## 1 Introduction

Solid polymer electrolytes (SPEs) are one promising option for replacing the liquid electrolyte in an all-solid-state battery (ASSB), resulting in a safer battery storage option. Moreover, SPEs offer high energy density due to their reduced packing size, mechanical flexibility and strength, easy thin-film fabrication, high capacity that only fades slowly, excellent interfacial contact with electrodes, and a longer lifespan.^1–3^ Among various ion-conducting polymers, conductive salt containing poly­(ethylene oxide) (PEO) stands out for its mechanical flexibility and high solubility of Li^+^-ions. However, these polymers lack mechanical stability, exhibit low ionic conductivity at room temperature, and have a narrow thermal and electrochemical stability window.^4^ To address these issues, we blend the good ion-conducting PEO with polyacrylonitrile (PAN) to act as a rigid backbone and nonconductive filler.^5^ Due to PAN’s enhanced thermal stability and its high melting point of 300 °C, the cell can operate at elevated temperatures when PEO is molten.^6^

Choi et al. were the first to use a PAN/PEO blend in gel polymer electrolytes, achieving a room-temperature conductivity of 1.2 × 10^–3^ S cm^–1^.^7^ A block copolymer of PAN and PEO is also suitable as a polymer electrolyte, and infrared analysis showed that the Li-ions coordinate to the plasticizer propylene carbonate’s C = O group, and the PEO’s C–O–C group, as well as a weak coordination to the PAN’s C≡N group.^8,9^

To maintain mechanical flexibility despite the rigid structure giving properties of PAN, we use electrospinning to produce our blend SPEs. A high voltage between a cannula connected to the polymer solution and a grounded collector generates an electric field. This causes charge separation, resulting in polymer fibers ranging from microns to nanometers in size.^10^ Compared to established SPE production methods, such as solution casting and hot pressing, electrospinning enables the production of tailored fiber morphologies from diverse materials, including core–shell and hollow structures. The resulting fibers are highly porous with large surface areas.^6^ Freitag et al. showed that electrospun PEO:LiBF_4_ SPEs exhibit decreased crystallinity compared to solution-cast SPEs with the same composition.^11^ Crystallinity is further suppressed by using plasticizers and lithium bis­(trifluoromethanesulfonyl)­imide (LiTFSI) instead of LiBF_4_.^12,13^ Zhang and Hsieh produced bicomponent PAN/PEO fibers for the first time and confirmed their phase separation using FTIR, NMR, DSC, TGA, and WAXS.^14^ Due to this phase separation and PEO’s higher ability to dissolve Li^+^-ions compared to PAN, PEO acts as the Li^+^-conducting polymer while PAN provides mechanical support.^8,15^ This combination of fibrous PAN/PEO electrolytes was further used by electrospinning PAN and PEO and soaking it in the liquid electrolyte,^16^ electrospinning only PAN and doctor-blading PEO:LiTFSI,^17^ PEO:SN:LiTFSI^18^ or PEO:PDMS:LiTFSI,^19^ respectively, into the fibrous mat, or electrospinning PAN with a ceramic and doctor-blading PEO:LiTFSI into the fiber-network.^20–22^ Kirchberger et al. were the first to use electrospun PAN/PEO blends with the conducting salt LiBF_4_ as polymer electrolytes. They proved that the immiscibility of the polymers in the fibers creates effective lithium-ion conduction pathways.^5^

In this work, we have prepared a series of highly flexible PAN/PEO SPEs with enhanced electrochemical stability by electrospinning all components in one step. The optimal ratio of the polymer blend of 50:50 wt % to the plasticizers succinonitrile (SN) and propylene carbonate (PC) to the conducting salt LiTFSI was found to be 18:9:1. The ionic conductivity can be tuned by 2 orders of magnitude by varying the humidity during electrospinning, as well as the precompression and measuring pressure, while maintaining a constant membrane composition. Scanning electron microscopy (SEM) images, differential scanning calorimetry (DSC), and thermogravimetric analysis (TGA) measurements confirm the thermal stability of the membrane. The SPEs demonstrate high electrochemical cycling stability in symmetrical Li|SPE|Li cells at 60 °C at current densities of 0.1 mA cm^–2^. The decomposition that occurred during cycling was evaluated by X-ray photoelectron spectroscopy (XPS) through postmortem analysis of the cycled SPE. Furthermore, solid-state nuclear magnetic resonance (NMR) spectroscopy of the conducting membrane indicates that moisture uptake affects the local chemical environment, as reflected in the ^1^H, ^13^C, and ^7^Li NMR spectra. Variable-temperature ^7^Li NMR measurements also revealed rapid local lithium-ion exchange dynamics and low activation energies. This study highlights the strong potential of these materials as electrolytes with high ionic conductivity and demonstrates the safe application of SPEs at elevated temperatures and the possibility of producing them in a single step.

## 2 Experimental Section

### 2.1 Preparation of Membranes

Polyacrylonitrile (PAN, Sigma-Aldrich, 150,000 g mol^–1^), Propylencarbonate (PC, Acros Organics) and Succinonitrile (SN, Thermo Scientific, purity > 99%) are weighed in a snap-on cap glass in the glovebox (O_2_ < 0.1 ppm, H_2_O < 2 ppm) and dissolved in 4.5 mL dimethyl sulfoxide (DMSO, VWR, less than 0.03% water). After complete dissolution, 4.5 mL acetonitrile (MeCN, MB-SPS; MBRAUN INTERTGAS-136 SYSTEME GmbH) and poly­(ethylene oxide) (PEO, Sigma-Aldrich, 300,000 g mol^–1^) are added after another. Lithium bis­(trifluoromethanesulfonyl)­imide (LiTFSI, Sigma-Aldrich, Caution! GHS Category 3 toxicity, handle with care) is added 3 h before electrospinning (ES). The respective amounts of the starting materials can be found in Table 1.

**Table 1:** Starting Material Quantities (0.0001 g Accuracy) for Electrospun Membranes of the Composition PAN/PEO: PC/SN: LiTFSI

	18:0:1	18:3:1	18:6:1	18:9:1	18:12:1	18:15:1	18:18:1
PAN	0.2500 g	0.2500 g	0.2500 g	0.2500 g	0.2500 g	0.2500 g	0.2500 g
PC		0.080 g	0.1603 g	0.2405 g	0.3207 g	0.4008 g	0.4810 g
		60 μL	130 μL	200 μL	266 μL	333 μL	400 μL
SN		0.0758 g	0.1516 g	0.2274 g	0.3032 g	0.3790 g	0.4547 g
PEO	0.2500 g	0.2500 g	0.2500 g	0.2500 g	0.2500 g	0.2500 g	0.2500 g
LiTFSI	0.1657 g	0.1657 g	0.1657 g	0.1657 g	0.1657 g	0.1657 g	0.1657 g

During electrospinning outside the glovebox at a custom-built electrospinner, described in the literature by Freitag et al., a voltage of 20 kV is applied between the blunt cannula and the grounded ring collector.^11^ Caution! Electrospinning involves high voltage. Do not approach the equipment while it is operating. The distance between the tip and the collector is 20 cm, and the feed rate of the stirred syringe is 4.0 mL h^–1^. For PAN membranes, 0.5 g PAN was dissolved in 7 mL dimethylformamide (DMF, Thermo Fisher Scientific, purity 99.8%) and electrospun at a voltage of 18 kV, with a tip-collector distance of 15 cm, and a feed rate of 2.3 mL h^–1^. After evaporation on air for approximately 2 h, the produced membrane is dried *in vacuo* and stored in the glovebox.

### 2.2 Materials Characterization

Scanning electron microscopy (SEM) images were obtained with a JSM-IT200 InTouchScopeTM from JEOL on a graphite sample holder at an acceleration voltage of 10.0 kV. The crystallinity of the samples was characterized by X-ray powder diffraction by a Stoe STADI P-Diffractometer using a Ge(111)-monochromator for Cu K_α_ (λ = 1.54056 Å) and a Dectris MYTHEN DSC 1 K solid-state detector. A sample disc was punched in the glovebox and measured airtight in Scotch Magic TapeTM8–1933R8 in a flat-bed sample holder. Thermal analysis was measured by differential scanning calorimetry (DSC) and thermogravimetric analysis (TGA). For DSC, 10 mg of the sample was sealed in an aluminum crucible in the glovebox and measured with a Netzsch Maia DSC 200 F3 between 123 and 523 K with a 10 K min^–1^ heating rate under continuous nitrogen flow. The second heating cycle was used for analysis. TGA experiments were performed using a TGA device (Mettler Toledo, Switzerland). Sapphire crucibles (70 μL, Mettler Toledo, Switzerland) were filled with the sample (approximately 70–100 mg) in the glovebox. After the sample insertion, the TGA setup was flushed with argon (100 mL min^–1^) at 25 °C to remove any residual ambient atmosphere. The sample was heated from 25 to 150 °C at 5 K min^–1^, with an argon flow rate of 60 mL min^–1^ and held at 150 °C for 30 min afterward. A Brukeroptics Senterra Spectrometer obtained *Raman* spectra with a 785 nm laser, 5% power, 5 s excitation time, and 10 accumulations. Liquid NMR was recorded on a Bruker Avance Ultrashield 400 MHz or a Bruker Avance Ultrashield 500 MHz Cryo system spectrometer. The signals of ^1^H spectra were referenced to the signal of the used, not dried, deuterated solvent d^6^-DMSO (2.50 ppm). Chemical shifts are given in δ values in parts per million (ppm). The spectra were evaluated using the program MestReNova.

### 2.3 Solid-State NMR Spectroscopy

Solid-state ^1^H, ^13^C, and ^7^Li NMR experiments were performed on a Bruker AVANCE NEO 500 spectrometer operating at a magnetic field strength (*B*_0_) of 11.75 T, using a 4 mm double-resonance (H/X) magic-angle spinning (MAS) probe. Dry membrane samples were prepared under argon to avoid the uptake of moisture due to their hygroscopic nature, and measurements were performed in a dry nitrogen stream, while the on air sample was handled under ambient conditions. The membrane samples were cut to fit the rotor dimensions, rolled, and inserted into 4 mm outer diameter zirconia (ZrO_2_) rotors. Kel-F caps were used to seal the rotors for room-temperature measurements, while Vespel caps were employed for variable-temperature experiments. All spectra were processed using Bruker Topspin 4.3.0, and plots were generated with Origin 2021.

(i) ^1^H NMR (ω_0_/2π = 500.26 MHz): Experiments were carried out using a Bloch pulse sequence^23^ with a 4 μs π/2 pulse (ω_1_/2π = 62.5 kHz), an optimized recycle delay of 2 s, and 16 coadded transients. The MAS rate was set to 14 kHz. The ^1^H chemical shifts (δ) were calibrated against a secondary reference, adamantane, assigning δ­(^1^H) = 1.85 ppm, referenced to the primary reference, tetramethylsilane (TMS) at δ­(^1^H) = 0 ppm.
(ii) ^13^C Cross-Polarization (CP) MAS NMR (ω_0_/2π = 125.80 MHz):^24^ For ^1^H excitation, a 4 μs π/2 pulse (ω_1_/2π = 62.5 kHz) was applied. Cross-polarization to ^13^C was achieved under Hartmann–Hahn^25^ matching conditions using a shaped pulse with a contact time of 2 ms, a recycle delay of 3 s, and 2048 coadded transients. Data were acquired at room temperature under MAS at 14 kHz. ^13^C chemical shifts were referenced to the high-frequency peak of adamantane at δ­(^13^C) = 38.56 ppm, relative to TMS at δ­(^13^C) = 0 ppm.
(iii) ^7^Li (ω_0_/2π = 194.42 MHz): Experiments were performed using a 4 μs Bloch decay (π/2) pulse (ω_1_/2π = 62.5 kHz). Spectra were acquired under MAS with a spinning frequency of 14 kHz, a 2s recycle delay, and 16 coadded transients. ^7^Li NMR spectra were referenced to a 1 M LiCl aq. solution, set at δ­(^7^Li) = 0 ppm.
(iv) ^1^H and ^7^Li Variable Temperature (VT) NMR: VT ^1^H NMR (295 to 363 K) and ^7^Li NMR (240 to 310 K) spectra were acquired using a 4 μs Bloch decay (π/2) pulse (ω_1_/2π = 62.5 kHz) with a 2 s recycle delay and four coadded transients under MAS (10 kHz) conditions. Sample temperature was controlled using a Bruker VT unit and corrected for frictional heating and instrument calibration using methylammonium lead chloride (CH_3_NH_3_PbCl_3_) powder.^26^

Spin–lattice relaxation times (*T*_1_) for both ^1^H and ^7^Li were determined using an inversion recovery sequence (π–τ_D_–π/2–acquisition), where τ_D_ is the variable delay. The *T*_1_ values were extracted by fitting the signal intensities to a single-exponential recovery function: *A*(*t*) = *A*(∞)­(1–2 $e^{-\left(t/T_{1}\right)})$ , where *A*(*t*) and *A*(∞) represent the NMR peak areas at time *t* and at equilibrium, respectively.

### 2.4 Electrochemical Characterization

The conductivity of the samples was measured via potentiostatic electrochemical impedance spectroscopy (PEIS). In the glovebox, a membrane disc (diameter 10 mm) is mounted between stainless-steel electrodes in an rhd TSC standard battery cell. A constant pressure of 0.62 MPa is applied by a spring (spring constant 32.6 N mm^–1^) if not stated otherwise. The measurement was operated by a Metrohm Autolab B.V. PGSTAT204 potentiostat in a temperature range of 293 K–328 K in 5 K steps, at an amplitude of 20 mV, in a frequency range of 10^6^–10^–1^ Hz. A pressure of up to 2.1 MPa was applied in the rhd cell with the same measurement setup by tightening a screw, compressing the spring. At the pressure of 12.7 MPa, a membrane disc (diameter 8 mm) is mounted between stainless-steel electrodes in an ASC-T cell (Sphere Energy, Germany) while applying pressure by a firmer spring (167 N mm^–1^). The system is measured with the same parameters.

The temperature sequence of a heating and a cooling cycle is repeated, and the fourth cycle is used for analysis. Subsequently, the thickness of the sample was measured by a Holex micrometer screw (0–25 mm, 0.001 mm accuracy). The data were analyzed using the RelaxIS3 software and an equivalent circuit model including a resistor and a constant phase element (R/Q) in parallel and another constant phase element in series ((R/Q)-Q)). Chronoamperometry was measured in the same setup at 50 mV, 100 mV, and 150 mV.

Electrochemical stability was characterized in an rhd TSC standard battery cell at a pressure of 0.62 MPa using a Biologic VMP3 potentiostat. A Li|SPE|Stainless steel (SS) cell at 25 °C was cycled between open circuit voltage (OCV) and 5.5 V vs Li^+^/Li at 0.1 mVs^–1^ to obtain the oxidative stability window. A new cell was cycled between OCV and 0 V vs Li^+^/Li at 0.1 mVs^–1^ to obtain the reductive stability window.

The lithium-ion transference number was determined in a symmetric Li|SPE|Li cell setup at 60 °C through DC polarization by analyzing the current and interfacial impedance change. The value of the Li^+^ transference number $t_{Li^{+}}$ was calculated by the Bruce-Vincent-Evans method using the following equation:

$$t_{Li^{+}}=\frac{I_{\mathrm{steadystate}}\left(\Delta V-I_{0}R_{Int,0}\right)}{I_{0}\left(\Delta V-I_{\mathrm{steadystate}}R_{Int,\mathrm{steadystate}}\right)}$$

where Δ*V* is the applied voltage of 10 mV, *R*_Int,0_ and *R*_Int,steadystate_ are the interface resistance values before and after DC polarization, respectively, determined by PEIS, and *I*_0_ and *I*_steadystate_ are the current values at the initial and steady state. To another symmetric cell at 60 °C, a current density between 0.05 and 1.4 mA cm^–2^ was applied in 0.1 mA cm^–2^ steps until the potential limit of 3.9 V to determine the critical current density. A current density of 0.1 mA cm^–2^ was applied to a symmetric Li|SPE|Li cell (galvanostatic plating/stripping) until end-of-life at 60 °C.

### 2.5 X-ray Photoelectron Spectroscopy

After the end-of-life of the galvanostatic plating/stripping experiment, the cell was disassembled, and the SPE was analyzed by XPS (Axis, Supra, Kratos, UK). Therefore, LiTFSI, a noncycled SPE and the end-of-life SPE (called cycled) were dried at 70 °C *in vacuo* overnight. The LiTFSI powder was pressed into a pellet, and the two SPEs (cycled/noncycled) were mounted on copper tape onto an inert sample holder inside the glovebox. The sample holder inside the inert transfer device was then flanged to the XPS antechamber (flexi lock). The sample was kept in the antechamber until a pressure of ∼1 × 10^–7^ Torr was reached and then transferred to the sample analysis chamber, where the pressure was always kept below ∼2 × 10^–8^ Torr during the whole measurement period. The excitation energy was provided with a monochromatic Al K_α_ source (1486.6 eV), using an emission current of 15 mA with a spot size of roughly 700 μm × 300 μm. The measurement procedure consisted of recording several different scans: 1) 2 survey scans from −5 to 1200 eV, with a 1.0 eV step size and a 200 ms dwell time; 2) 3 scans of the S 2p region from 150 to 178 eV, with a 0.1 eV step size and a 500 ms dwell time; 3) 3 scans of the C 1s region from 270 to 303 eV, with a 0.1 eV step size and a 500 ms dwell time; 4) 3 scans of the N 1s region from 385 to 415 eV, with 0.1 eV step size and a 500 ms dwell time; 5) 3 scans of the O 1s region from 520 to 548 eV, with a 0.1 eV step size and a 500 ms dwell time; 6) 3 scans of the F 1s region from 670 to 700 eV, with a 0.1 eV step size and a 500 ms dwell time. For the binding energy correction, the C 1s of the LiTFSI sample was referenced to the adventitious carbon C 1s peak (284.8 eV). Subsequently, the binding energy of the SPE samples was corrected by the F 1s peak (688.5 eV) of LiTFSI. For the detail-scan spectra, a Tougaard background was subtracted. Following, a min-max normalization was performed. Then the spectra were fitted with a superposition of a Gaussian (70%) and Lorentzian (30%) shape function (GL30). For the S 2p spectra, the S 2p_3/2_ and S 2p_1/2_ were fitted in a constrained manner with a difference in binding energy of 1.16 eV, and the S 2p_1/2_ had half the peak area of the S 2p_3/2_.

## 3 Results and Discussion

The production process of SPEs is shown in Figure 1a. First, PAN, PC, and SN were dissolved in DMSO. Then, MeCN and PEO, as well as LiTFSI, were added after that. Electrospinning the homogeneous solution enables, in principle, a roll-to-roll production (Figure 1a). The membrane sits on air for a specific evaporation time before removing the solvent *in vacuo*. After drying, the obtained porous membrane is free-standing, highly flexible, and mechanically stable, as illustrated in Figure 1b,c. The SPE can easily be bent and folded, both with and without the addition of a plasticizer. The indicated compositions of 18:X:1 reflect the molar ratios of PEO:SN:LiTFSI and PAN:PC:LiTFSI, respectively, while the polymers PAN and PEO are mixed in the weight ratio of 50:50 (Table 1). This ratio was optimized by Kirchberger et al.^5^ SEM images of an 18:9:1 membrane illustrate a fiber network with fiber diameters between below one μm and a few μm, as well as very few fiber bundles and beads. The fibers are randomly oriented without propagating in a specific direction (Figure 1d).

**Figure 1:**
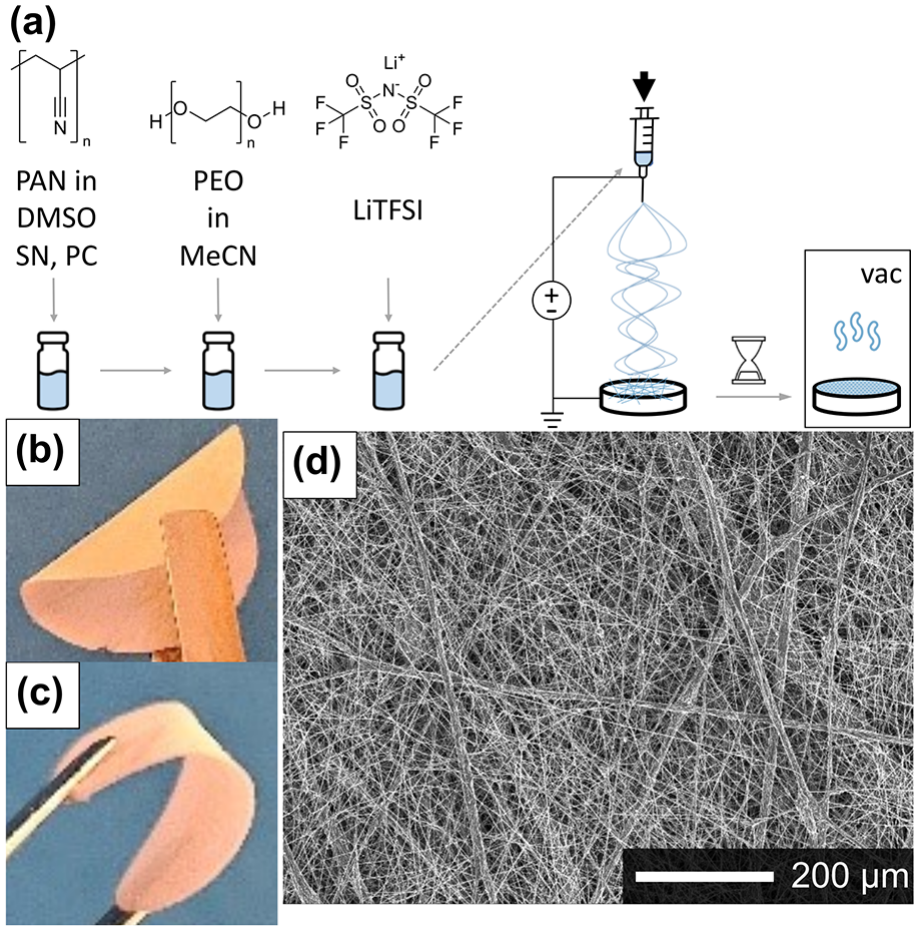
(a) Schematic illustration of the single-step production of an SPE membrane, (b) Image of a plasticizer-free and (c) a plasticizer-containing bent electrolyte membrane, and (d) SEM image of an 18:9:1 SPE.

*Raman* spectroscopy was used to evaluate the bonding behavior in the SPE compared to the electrospun polymers PEO and PAN alone, as well as an electrospun blend of both (Figure 2a). The bands at 800–900 cm^–1^, the vibration modes of the ether oxygen, provide insight into the interaction between lithium and PEO. While the two peaks in this region are sharp and intense for electrospun PEO, their intensity decreases and their peak shape broadens, respectively, for electrospun PAN/PEO and even more so for the lithium- and plasticizer-containing SPE. This decrease in intensity indicates a loss of the crystalline PEO phase in the plasticized polymer electrolyte. However, the shift of the C–O–C band from 860 cm^–1^ in PEO to 870 cm^–1^ in the SPE results from a polarization of the ether bond due to coordination to a Li^+^-ion.^27,28^ These findings suggest that Li^+^ binds to the ether units of PEO in the SPE. Additionally, the band at 740 cm^–1^ in the SPE can be assigned to the S–N–SCF_3_ vibration mode of the conducting salt LiTFSI.^29^ The mode at ∼2200 cm^–1^ can be attributed to the CN stretch of PAN and does not change for the PAN-containing membranes. This suggests that the nitrile group of PAN is not involved in lithium-ion conduction. These results support the hypothesis that PAN mechanically supports PEO but is not involved in ionic conduction. The bands at 2800–3000 cm^–1^ represent the C–H stretches in the polymers and plasticizer.^27^

**Figure 2:**
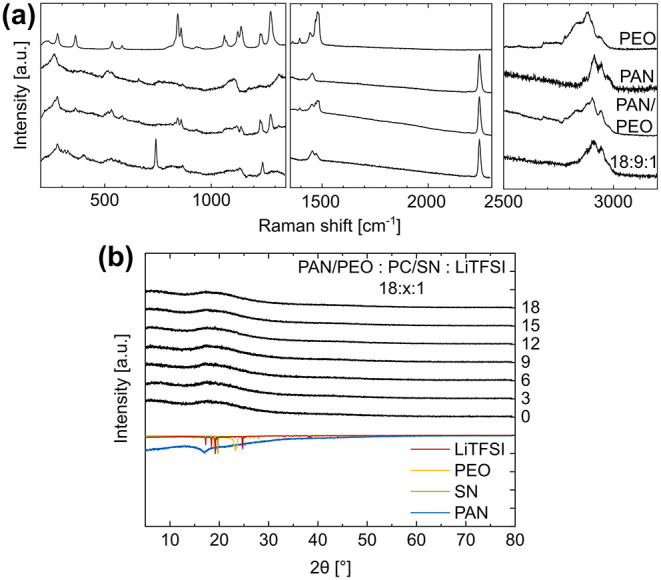
(a) *Raman* spectroscopy of an 18:9:1 SPE compared to the electrospun polymers or polymer blend alone and (b) X-ray powder diffraction of SPEs with increasing plasticizer content compared to their crystalline primary materials.

The XRD results show that the membranes are fully amorphous with and without plasticizers (Figure 2b). While electrospun PEO:SN:LiTFSI SPEs exhibit crystalline reflections, blending PAN into PEO decreases crystallinity.^12^ Furthermore, blending PAN into PEO enables the production of such high plasticizer contents with electrospinning. In membranes containing only PEO, SN, and LiTFSI, produced by Walke et al., the maximum electrospinnable plasticizer content was 25 wt % at a PEO:SN:LiTFSI composition of 36:8:1.^12^ At a higher content, the fibers fused during electrospinning. This work demonstrates the production of plasticizer contents of up to 58 wt %.

Although increasing the plasticizer content does not affect crystallinity, the ionic conductivity at room temperature increases by 1 order of magnitude, reaching 1.6 × 10^–5^ S cm^–1^ with a PAN/PEO:PC/SN:LiTFSI composition of 18:12:1, compared to no plasticizer at a composition of 18:0:1 (Figure 3a, blue). Additionally, a moderately higher plasticizer content simplifies the producibility and flexibility of the SPE. However, if the plasticizer content is too high, the conductivity drops again, as observed for the compositions of 18:12:1 and 18:18:1. The best-producible and measurable membrane had a composition of 18:9:1, so we investigated this composition further. However, most of the plasticizer PC and fractions of SN evaporate during electrospinning and drying, as found by Spranger et al.^30^ This was confirmed in this work by liquid NMR in d^6^-DMSO (Figure S1 and Table S2).

**Figure 3:**
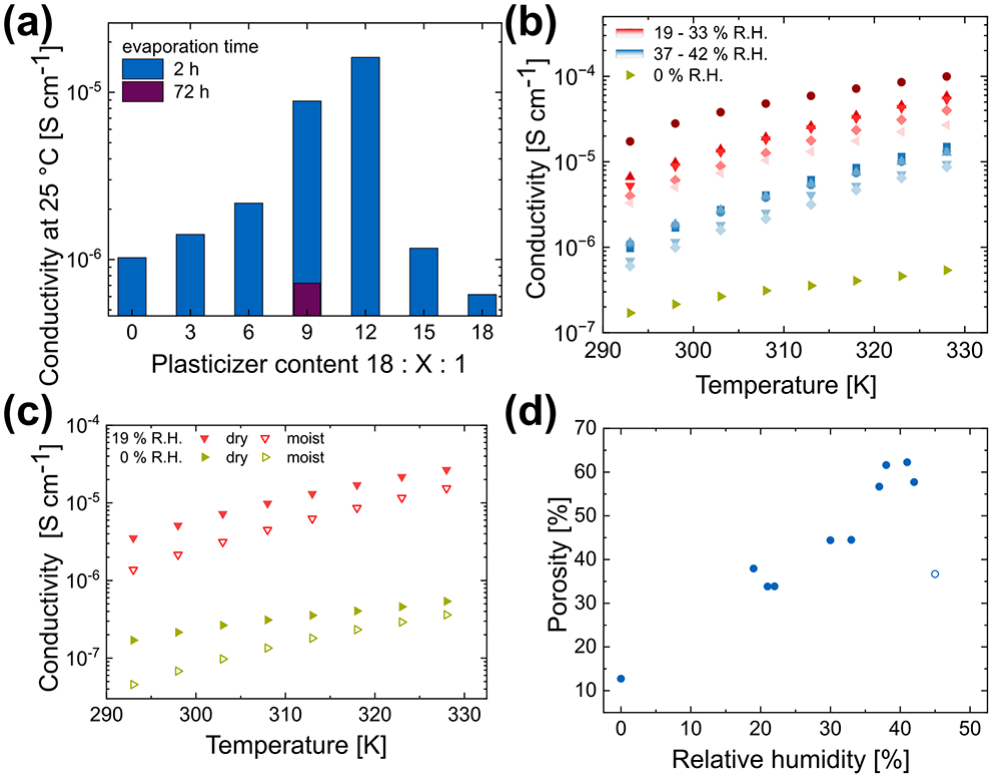
Conductivity of SPEs depending on (a) the plasticizer content and evaporation time. Conductivity of SPEs of the composition PAN/PEO:PC/SN:LiTFSI 18:9:1 depending on (b) relative humidity (R.H.) during electrospinning, and (c) moisture content. (d) Correlation between relative humidity and calculated porosity for normally dried membranes (filled circles) and a pressed membrane before drying (empty circle).

Besides the plasticizer content, the drying of the electrospun membrane influences the conductivity. If the membrane is dried *in vacuo* immediately after electrospinning, the rapid evaporation of the remaining solvent can cause the fibers to tear. To avoid this, the membrane is stored on air to allow the solvent to evaporate, which is called the evaporation time. While an evaporation time of 72 h leads to a conductivity drop of more than an order of magnitude, an evaporation time of one to 2 h strikes a balance between ripping and good conductivity (Figure 3a, purple vs. blue). Therefore, all subsequent membranes were electrospun with a PAN/PEO:PC/SN:LiTFSI composition of 18:9:1, and an evaporation time of 1 to 2 h.

In addition to using the proper drying method, it is crucial to monitor and control production parameters. As Figure 3b shows, relative humidity (R.H.) during electrospinning affects the ionic conductivity by 2 orders of magnitude. The best-performing membrane, spun at an optimal relative humidity of 30%, exhibits an ionic conductivity of 1.0 × 10^–4^ S cm^–1^ at 55 °C and 2.8 × 10^–5^ S cm^–1^ at 25 °C. This meets the requirement of 10^–4^ S cm^–1^ for normal use and application of SPE-based batteries.^31^ Its electric conductivity, as determined by chronoamperometry, is 10^–10^ S cm^–1^ at 25 °C. All membranes spun at a higher or lower relative humidity exhibit a lower ionic conductivity. The membrane with the lowest conductivity of 5.4 × 10^–7^ S cm^–1^ at 55 °C and 2.1 × 10^–7^ S cm^–1^ at 25 °C was electrospun in a dry room with a dew point of −45 °C, where the relative humidity is less than 0.5%. All membranes exhibit an activation energy ranging from 0.35 to 0.64 eV, as determined from the Arrhenius plot (Figure S2 and Table S1).

One possible reason for the deviation in R.H.-dependent ionic conductivity might be the moisture accumulation in the membranes. In this case, the increased conductivity would be due to a higher water content. To test this hypothesis, a well-conducting membrane from the red region in Figure 3b (19%–33% R.H.) and the membrane with the worst conductivity in the green region (0% R.H.) were enriched with moisture. First, the ionic conductivity of a dried membrane disc was measured. Then, the same discs were exposed to an ambient atmosphere (50% R.H.) for 4 days to allow moisture to accumulate, and their ionic conductivity were measured again. The results show a decrease in ionic conductivity after moisture accumulation (Figure 3c). Solid-state ^1^H and ^13^C­{^1^H} cross-polarization magic-angle spinning (CPMAS) NMR spectroscopy of the dry membrane before and after moisture exposure indicates changes in ^1^H and ^13^C chemical environments, but no change in the backbone structures. (Figure S4) A slight increase in the water signal around 4 ppm is observed in the ^1^H NMR spectrum of the air-exposed sample, indicating moisture absorption. However, ^7^Li MAS NMR reveals a change in the local Li environment. The LiTFSI peak of the moisture-exposed SPE shifts by 0.32 ppm toward higher frequencies, reaching −0.95 ppm compared to the dry sample at −1.27 ppm as observed previously (Figure S5).^32^ This peak can also be assigned to the chemical shift of LiF (−1.0 ppm), which forms alongside hydrofluoric acid during the hydrolytic decomposition of LiTFSI.^33,34^ Upon drying, the ^7^Li resonance completely shifts back to a lower frequency, indicating changes in the local lithium environment. As LiF does not evaporate during the drying process, we propose the formation of a LiTFSI-water adduct. This adduct is likely less mobile, which could contribute to a reduction in ionic conductivity. To sum up, after exposure to moisture, the changed Li-environment decreases the Li^+^-conductivity.

Another possible reason for the R.H.-dependent deviation may be a change in microstructure due to the modified evaporation behavior. According to Kim et al., PEO primarily conducts Li^+^-ions while PAN acts as a rigid backbone.^15^ Since PEO is soluble in acetonitrile (boiling point: 82 °C) and PAN is soluble in dimethyl sulfoxide (boiling point: 189 °C), each polymer solidifies as its respective solvent evaporates. Relative humidity during electrospinning influences solvent evaporation behavior and, therefore, the SPE microstructure. Figure 3a shows another indication of this hypothesis: conductivity depends on the drying method.

An easily characterizable change in microstructure is the porosity. Therefore, we calculated the porosity of a membrane disc from its dimensions, weight, and composition after the potentiostatic electrochemical impedance spectroscopy (PEIS) measurement. We then correlated the porosity to the relative humidity during electrospinning (Figure 3d).

$$\mathrm{Porosity}=1-\frac{V_{\mathrm{reactants}}\left[cm^{3}\right]}{V_{\mathrm{membrane}}\left[cm^{3}\right]}=1-\frac{\underset{i}{\sum }\frac{m_{i}\;[g]}{\rho_{i}\left[g,cm^{-3}\right]}\cdot \frac{m_{\mathrm{membrane}}\left[g\right]}{m_{\mathrm{reactants}}\left[g\right]}}{\pi \cdot r^{2}\left[cm^{2}\right]\cdot t\left[cm\right]}$$

Porosity decreases with decreasing relative humidity, with one exception. This exception (Figure 3d, empty circle) was densified by rolling before drying and, therefore, shows a lower porosity than membranes with similar relative humidity. Lower porosity correlates loosely with increasing ionic conductivity, except for the membrane electrospun in the dry room. This membrane shows low room-temperature ionic conductivity of 2.15 × 10^–7^ S cm^–1^ at a porosity of 12.7% (Figure S3). Its porosity is comparable to that of a solution-cast SPE of the same composition, where the missing fiber structure leads to a comparable low ionic conductivity of 2.5 × 10^–7^ S cm^–1^. Therefore, we hypothesize that the fibers fused during electrospinning in the dry room, as observed in SEM images. This fusion leads to a collapse in the fiber structure and a decrease in ionic conductivity (Figure S6).

Pressure was applied to the membranes to decrease their porosity artificially. This was done either by precompression at room temperature before the PEIS measurement, denoted as fabrication pressure in the literature, or by increasing the cell pressure during the measurement, called operation pressure in the literature.^35^ In the latter case, the membrane is heated to 55 °C at the applied operation pressure during the measurement. Before applying any pressure, the fiber structure is fully intact, and the membrane appears entirely white and homogeneous (Figure 4a, left). If the membrane is pressed, the white membrane gets translucent scratches. The more pressure is applied, the more parts of the membrane become translucent as the fiber structure collapses, and the membrane takes on a solution-cast-like morphology. The 102 μm thick membrane becomes thinner during pressing and reaches approximately one-third of its initial thickness after applying 62.5 MPa. Porosity decreases from 69% to 43%.

**Figure 4:**
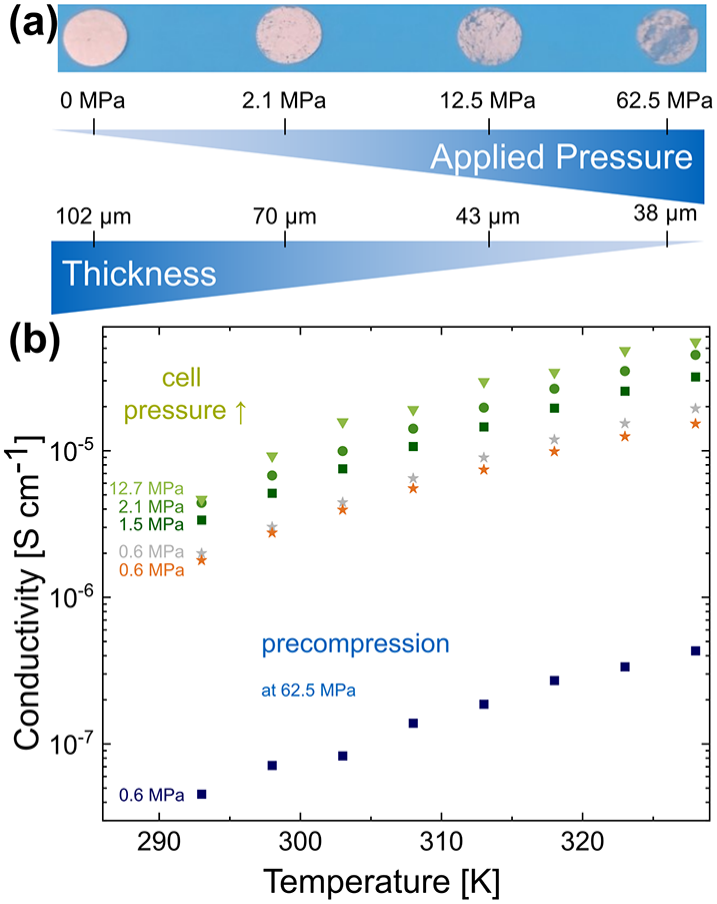
Mechanical Stability: (a) images of membrane discs and their thickness before any pressure application and after precompressing the SPEs at the indicated pressures. (b) Temperature-dependent conductivity and its change due to applied pressure either during a PEIS measurement in the cell (green) or before a PEIS measurement via precompression (blue) compared to a nonprecompressed membrane measured at a cell pressure of 0.6 MPa (orange). For checking, after applying a cell pressure of 2.1 MPa to the membrane, it was measured again at 0.6 MPa (gray).

The conductivity of the precompressed SPEs decreases if high pressure is applied before the measurement (Figure 4b, blue). The ionic conductivity of an SPE precompressed at 62.5 MPa decreases by 1.5 orders of magnitude compared to a nonprecompressed membrane (Figure 4b, blue vs orange). However, the porosity of the precompressed membranes is lower. The applied pressure destroys the fiber structure, as also seen in SEM images of the translucent part of the membrane (Figure S8); therefore, the membrane conducts like a solution-cast one, which was also observed for electrospun only-PEO membranes.^11^ Unlike those electrospun PEO membranes, our PAN/PEO membranes are form-stable under pressure and retain their original shape. If a small precompression pressure is applied, the ionic conductivity is slightly higher or lower than that of the uncompressed membrane, depending on which part of the membrane is measured (Figure S9). Therefore, one must be careful comparing different membrane parts with one another, as the ionic conductivity varies within half an order of magnitude.

However, the conductivity increases when the operating pressure is applied consistently during the PEIS measurement, in which the membrane is heated to 55 °C (Figure 4b, green). Increasing the cell pressure from 0.6 to 2.1 MPa doubles the conductivity. An extensively higher cell pressure of 12.5 MPa does not significantly enhance conductivity. When the SPE, which was pressed at 2.1 MPa and 55 °C during the measurement, is measured again at the standard cell pressure of 0.6 MPa, the conductivity remains nearly unchanged (Figure 4b, gray stars). After each measurement, the thickness was measured, and the cell was reassembled to exclude errors related to thickness from the conductivity calculations. Additionally, all orange/gray/green data points were measured with the same membrane disc to exclude inhomogeneity-related errors within the membrane.

Based on these findings, we draw three major conclusions. First, strong precompression at room temperature worsens the conductivity of an SPE due to fiber destruction. Second, conductivity can be enhanced by up to half an order of magnitude by increasing the cell pressure to 2.1 MPa. Third, applying much higher cell pressure does not further enhance conductivity. Together, these findings provide a significant advantage in using electrospun PAN/PEO solid electrolytes. The fabrication for and the operation in ASSBs is straightforward. Dried membranes do not need to be precompressed before assembly with the anode and cathode. Additionally, the stack pressure, which must be constantly applied to an ASSB, is relatively low at 2.1 MPa. Low pressure is crucial for commercializing ASSBs and their application in battery electric vehicles. According to Zhang et al., acceptable operation pressure for the original equipment manufacturer is 2 MPa or lower, a threshold achieved in this study.^35^

A long-term PEIS measurement conducted over 18 heating/cooling cycles at 0.6 MPa confirms the SPE’s mechanical stability. The ionic conductivity remains constant over time at the applied pressure (Figure S7).

In addition to mechanical stability, blending PAN into PEO promises to increase the thermal stability due to PAN’s high melting point. DSC analysis reveals that the glass transition temperature of the SPE is −43 °C, which confirms its amorphous nature at room temperature. The analysis also reveals endothermal features at 30 and 149 °C (Figure 5a). The tiny peak at 30 °C is due to the partial melting of small fractions of crystalline PEO. By comparison, electrospun PEO:SN:LiTFSI membranes melt between 40 and 50 °C. However, the melting onset is often found at around 30 °C.^12^ PEO powder and an electrospun PAN/PEO membrane without plasticizer or LiTFSI show a melting point of 66–67 °C (Figure S10). Thus, adding plasticizer and salt lowers the melting point of PEO. Additionally, the melting enthalpy, the integral of the melting peaks, decreases drastically from pure PEO to electrospun PAN/PEO to PAN/PEO with plasticizer and LiTFSI. This supports the hypothesis that PAN and the other additives stabilize PEO. By comparing the DSC spectrum to those of pure PAN powder and electrospun PAN, the peak at 149 °C can be assigned to tiny fractions of crystalline PAN (Figure S10). The PAN fibers exhibit a narrower DSC feature than the PAN powder. We assume that the PAN fibers are oriented due to the electric field in electrospinning, and that this results in a more localized effect in the DSC, temperature-wise. No effects of DMSO, MeCN, or water were found in DSC measurements; MeCN and DMSO do not form an azeotrope.^36^ In summary, the DSC shows the individual features of both polymers, confirming their matrix-like coexistence.

**Figure 5:**
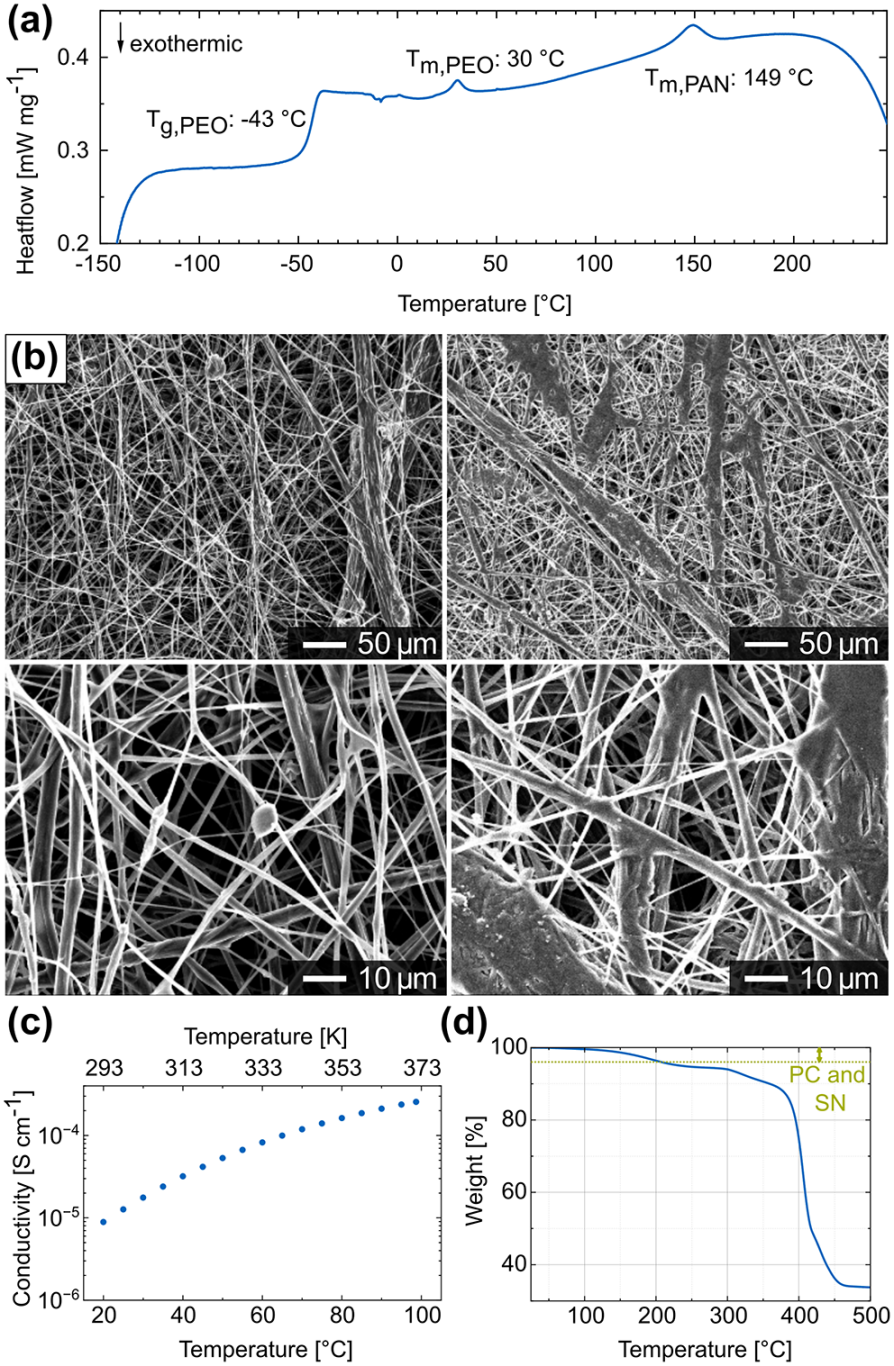
Thermal stability. (a) DSC curve of an SPE membrane with a composition of 18:9:1, (b) SEM images before (left) and after (right) heating to 100 °C at different magnifications, (c) temperature-dependent ionic conductivity of an SPE membrane up to 95 °C as well as (d) TGA results of an SPE membrane with a composition of 18:9:1. The inset in (d) shows the decomposition/evaporation of the plasticizers PC and SN in the SPE, as calculated using the liquid NMR results (Table S2, SI).

Although PEO may melt at 30 °C in the SPEs, coelectrospinning with PAN preserves the fiber structure after heating. Figure 5b shows SEM images of an SPE membrane at different magnifications before (left) and after (right) heating to 100 °C, at a pressure of 0.6 MPa. While some fibers on the top surface that touched the pressing die are plastically deformed, most membrane fibers remain intact. This enables an application of the polymer membrane above the melting temperature of PEO without risking a shortening of the cell due to a molten separator. Impedance measurements up to these temperatures show an ionic conductivity of 8.2 × 10^–5^ S cm^–1^ at 60 °C and 2.3 × 10^–4^ S cm^–1^ at 95 °C (Figure 5c). The SPE shows no clear Arrhenius behavior in this temperature range (Figure S11).

Thermogravimetric Analysis (TGA) measurements reveal an insignificant weight loss below 100 °C, which originates from the evaporation of water during sample transfer and residual PC (Figure 5d). Subsequently, a weight loss occurs below 250 °C due to the decomposition and evaporation of SN, as previously reported by Zhang et al.^18^ The combined weight loss of PC and SN up to 250 °C, as determined by liquid NMR in d^6^-DMSO and found to be 3.98 wt %, does not precisely correspond to the experimental weight loss of 5.9 wt % (Table S2). Additional weight loss may be due to solvent residues and water residues. The latter occurs due to the noninert transfer to the TGA device because water is excluded with DSC measurements. Following that, a rapid weight loss begins at ∼300 °C due to the complete decomposition of PEO and the partial decomposition of PAN and LiTFSI.^19,21,37,38^ TGA confirms the membrane’s thermal stability and a possible application up to at least 100 °C, if not 250 °C.

To use SPEs in full cells, the electrochemical stability of the membrane should fall within the voltage range of the commercially available anode and cathode materials. This range extends from 0 V for a lithium metal anode to approximately 4.3 V for state-of-the-art commercial cathode materials.^31^ While PEO’s stability potentially does not exceed 4.0 V, our SPEs demonstrate an electrochemical stability window ranging from 0 V to greater than 4.5 V vs Li^+^/Li, as determined by cyclic voltammetry (CV) in Li|SPE|stainless steel (SS) cells (Figure 6a). This renders our SPE suitable for use with high-voltage cathodes, either as a separator between the anode and cathode or as a catholyte. The ion transport characteristics were further investigated by calculating the lithium transference number via the Bruce–Vincent–Evans method to 0.18 at 60 °C, which is in the range of polymer SPEs (Figure S12, SI).^39,40^ However, it is still considered low and can cause local polarization, resulting in uneven Li^+^ deposition.^31^

**Figure 6:**
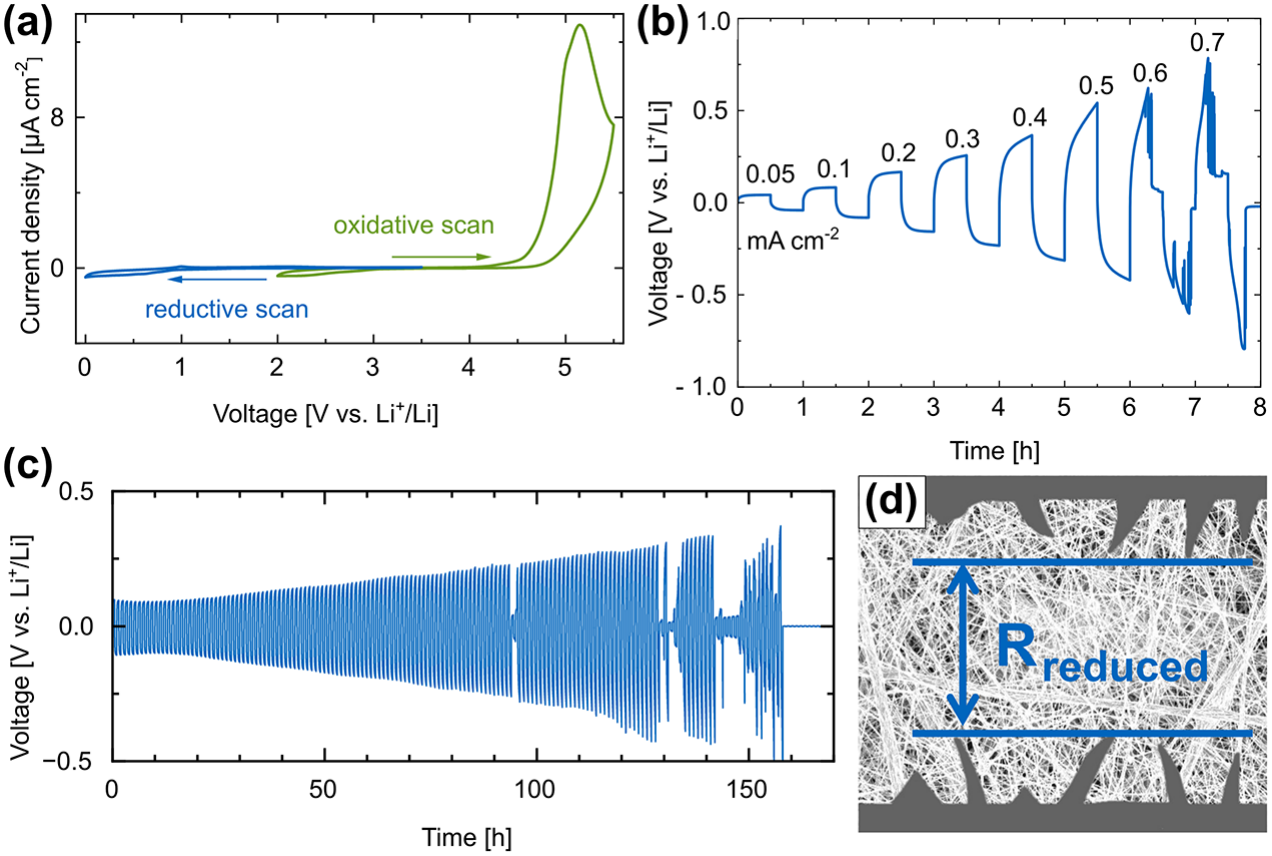
Electrochemical characterization of 18:9:1 membranes. (a) Electrochemical stability window determined by separate reductive and oxidative CV scans of a Li|SPE|SS cell at 25 °C, (b) critical current density measurements in a symmetrical Li|SPE|Li cell at 60 °C, (c) galvanostatic plating/stripping at 0.1 mA cm^–2^ in a symmetrical Li|SPE|Li cell at 60 °C (one full cycle in 1 h), and (d) schematic lithium dendrite formation in an SPE leading to a reduced resistance between the dendrites, adapted with permission from.^42^ Copyright 2021 Wiley.

Furthermore, our 18:9:1 SPE can withstand a current density of at least 0.5 mA cm^–2^ at 60 °C before degradation begins (Figure 6b). We assembled symmetrical Li cells to test long-term cycling stability and performed galvanostatic plating/stripping. At 0.1 mA cm^–2^ and a capacity of 0.05 mAh cm^–2^, the SPE can run for over 125 cycles or hours, respectively (Figure 6c).

Other PEO-based composite polymer electrolytes were able to perform galvanostatic plating/stripping longer without failure, for example, PEO with 1-butyl-2,3-dimethylimidazolium-bromide and LiTFSI at 0.1 mA cm^–2^ for 800 h, or an electrospun PAN-matrix with doctor-bladed PEO:LiTFSI at 0.5 mA cm^–2^ for 300 h.^17,41^ However, these systems are not fully comparable to our system, which is easier to fabricate in a scalable roll-to-roll one-step process. This was the first successful plating/stripping experiment of an electrospun PAN/PEO blend for application in ASSBs.

During Li^+^ plating/stripping, tiny dendrites, penetrating the membrane but not piercing through it, are forming, leading to a partial short circuit (Figure 6d).^42,43^ Due to the reduced resistance between the dendrites, the voltage drops. The dendrite regenerates with further Li^+^ shuttling until the cell dies after several partial short circuits. Over its lifespan, the overpotential increases linearly. This indicates an unstable solid electrolyte interface between the lithium and SPE, resulting in increased overall impedance.^17^

To understand the overpotential increase and the fading mechanism, we analyzed the lithium-SPE interface by X-ray photoelectron spectroscopy (XPS) after end-of-life, compared to a noncycled SPE sample and LiTFSI powder (Figures 7, S13–S15). Since no species could be identified that certainly remained constant over time (in terms of quantity) and would have been suitable for normalization, every single detailed spectrum was min-max normalized. In the F 1s spectra (left panels), LiTFSI and, most likely, its decomposition product LiF can be seen. Comparing the cycled and noncycled SPEs, more LiF at 684.7 eV (F 1s, green) relative to the LiTFSI feature at 688.5 eV (F 1s, red)^17,33,44^ evolved during cycling. The area ratio of F 1s of the LiTFSI peak to the LiF peak decreases from 91:9 of pure LiTFSI to 77:23 of the not-cycled sample to 70:30 of the cycled sample, indicating a decomposition of the LiTFSI during cycling.

**Figure 7:**
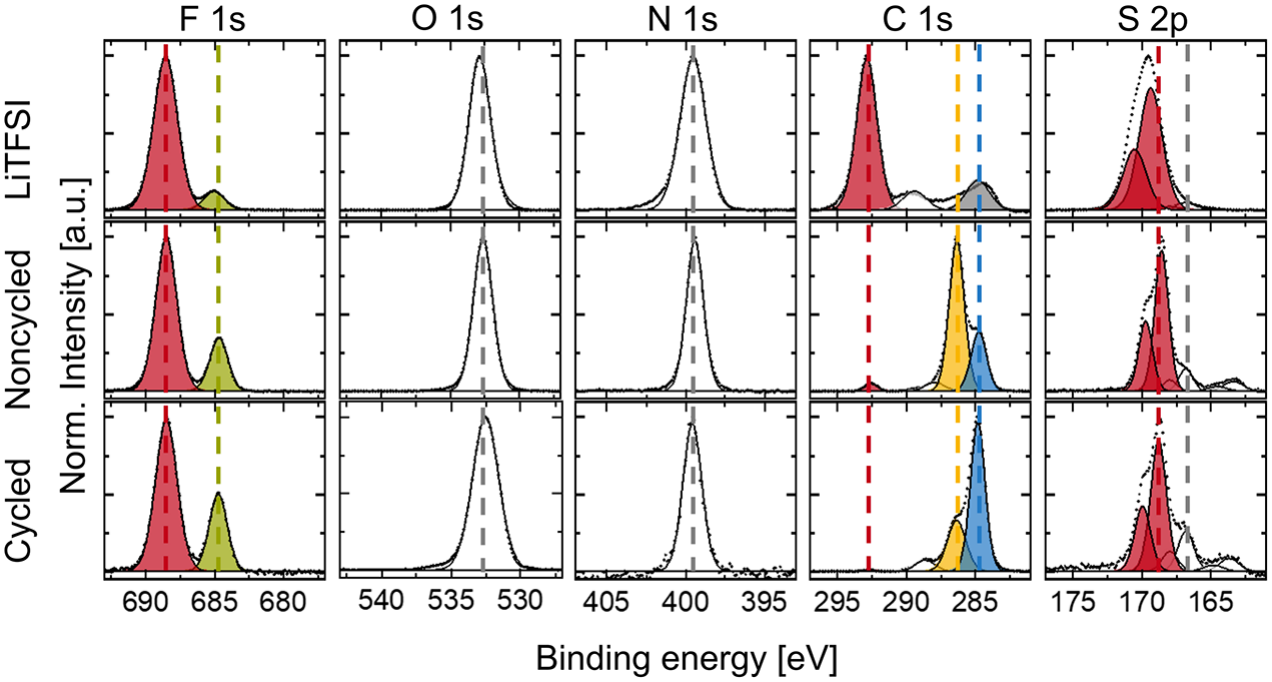
XPS detailed spectra of F 1s, O 1s, N 1s, C 1s, and S 2p of LiTFSI (top), a noncycled SPE (middle) and a SPE after lithium galvanostatic plating/stripping (bottom). The peaks reflect the species LiTFSI (red), LiF (green), ether and nitrile moieties of PAN and PEO (yellow), PAN’s CH_2_-moieties, adventitious carbon, as well as decomposition products of both polymers (blue), and adventitious carbon (gray).

The peak in the O 1s spectra (2nd from the left panels) at 532.5 eV, to which the PEO and the LiTFSI contribute, slightly broadens, indicating decomposition of the respective species. The N 1s peak (middle panels) originating from the PAN and the LiTFSI stays roughly constant in shape and binding energy before and after cycling. In the C 1s spectra (2nd from the right panels), for the LiTFSI, three peaks are observed one at 292.9 eV (C 1s, red) which is related to the LiTFSI itself, one at 289.4 eV (C 1s, white) which we would hypothesize is linked to a decomposition of LiTFSI and one at 284.8 eV (C 1s, gray) which we correlate to adventitious carbon. For the cycled and noncycled SPEs, four peaks could be observed. As for the pure LiTFSI sample, the peak at 292.7 eV (C 1s, red) is related to the LiTFSI. Interestingly, the intensity of this peak decreases for the cycled SPE compared to the noncycled SPE. In the noncycled SPE, this peak represents 3% of the total C 1s intensity (similar to the expected amount of 3.9% of the total C 1s intensity due to the composition of the SPE), while for the cycled SPE, this peak only resembles 0.6% of the total C 1s intensity. Within the assumption that no significant amount of carbon was introduced during the cycling experiment, this result indicates a significant decrease in LiTFSI concentration in the interphase between the Li metal and the SPE membrane on the membrane side, hence most likely forming a resistive interphase. As XPS is a surface-sensitive technique with an inelastic mean free path of roughly 2 to 3 nm for a polymer sample, we do not have more information about the thickness of the interphase layer or about the composition of the bulk. The peak at 288.6 eV (C 1s, white) could not be identified with absolute certainty; we hypothesized it originates from LiTFSI decomposition products, as a shift to lower binding energies resembles a higher electron density around the carbon atom, hence a reduced environment caused by lithium metal exposure. When comparing the cycled SPE and the noncycled SPE, this peak is clearly more pronounced after cycling. The third peak at 286.3 eV (C 1s, yellow) we assign to the PEO’s ether and PAN’s CN moieties. Its intensity with respect to the total intensity of the carbon signal decreases while the hydrocarbon peak at 284.7 eV (C 1s, blue) increases.^45,46^ The latter reflects PAN’s CH_2_-moieties and adventitious carbon in the noncycled sample. The increase of the fourth peak after cycling, we hypothesize, arises from the reduction of polymer species due to the contact with Li metal. In the S 2p spectra (right panels), for the spectrum of LiTFSI, two peaks appear at 168.6 and 169.7 eV corresponding to the S 2p_3/2_ and S 2p_1/2_ of LiTFSI. In addition, mainly for the noncycled and cycled SPE, there are at least two further species. The corresponding peaks of the S 2p_3/2_ are at 166.8 and 163.4 eV. Both species increase when comparing the cycled species to the noncycled with respect to the total S 2p intensity, indicating sulfur-containing decomposition products. The latter species at 163.4 eV would be in the range of elemental sulfur.^47^ In conclusion, the decomposition of the lithium salt LiTFSI and reduction of the polymers PEO/PAN support the hypothesis of an unstable interface formed during Li shuttling in the SPE.

To investigate the local Li-ion mobility in the dry membrane, variable-temperature nonspinning ^7^Li solid-state NMR spectra were recorded over a temperature range of 240 to 310 K. As shown in the stacked spectra in Figure 8a, the ^7^Li line width gradually narrows with increasing temperature. This temperature-dependent line narrowing is attributed to enhanced lithium-ion mobility at elevated temperatures, which leads to motional averaging of ^7^Li–^7^Li homonuclear dipolar interactions, thereby reducing the observed line width. The corresponding plot of the full width at half-maximum (fwhm) as a function of temperature is presented in Figure 8b. Two distinct plateau regions are observed in the temperature-dependent fwhm data. At low temperatures (*T* ≤ 250 K), the fwhm reaches a constant value of approximately 4 kHz, characteristic of the rigid-lattice regime, where Li-ion motion is negligible. In contrast, at higher temperatures (*T* ≤ 300 K), a second plateau appears, corresponding to the rapid motional narrowing. Here, the fwhm decreases significantly, reaching a minimum of ∼0.5 kHz, indicating substantial line narrowing due to rapid lithium-ion dynamics and effective averaging of the dipolar interactions. The onset temperature (*T*_onset_) for line narrowing, associated with the initiation of lithium-ion mobility, allows for an estimation of the activation energy (*E*_A_) for the dynamic process using the empirical Waugh-Fedin relation:^48^

$$E_{A}(kJmol^{‐1})=0.156\times T_{onset}[K]$$

where *T*_onset_ is defined as the temperature at which the line width decreases by half the difference between the rigid-lattice and motional-narrowing limits, i.e., $\frac{1}{2}\times$ (fwhm_rigid lattice_ – fwhm_motional narrowing_). Based on this criterion, an onset temperature of approximately 267 K is identified, yielding an estimated activation energy of 42 kJ mol^–1^ for Li-ion motion. This lies in reasonable agreement with the activation energies measured by PEIS.

**Figure 8:**
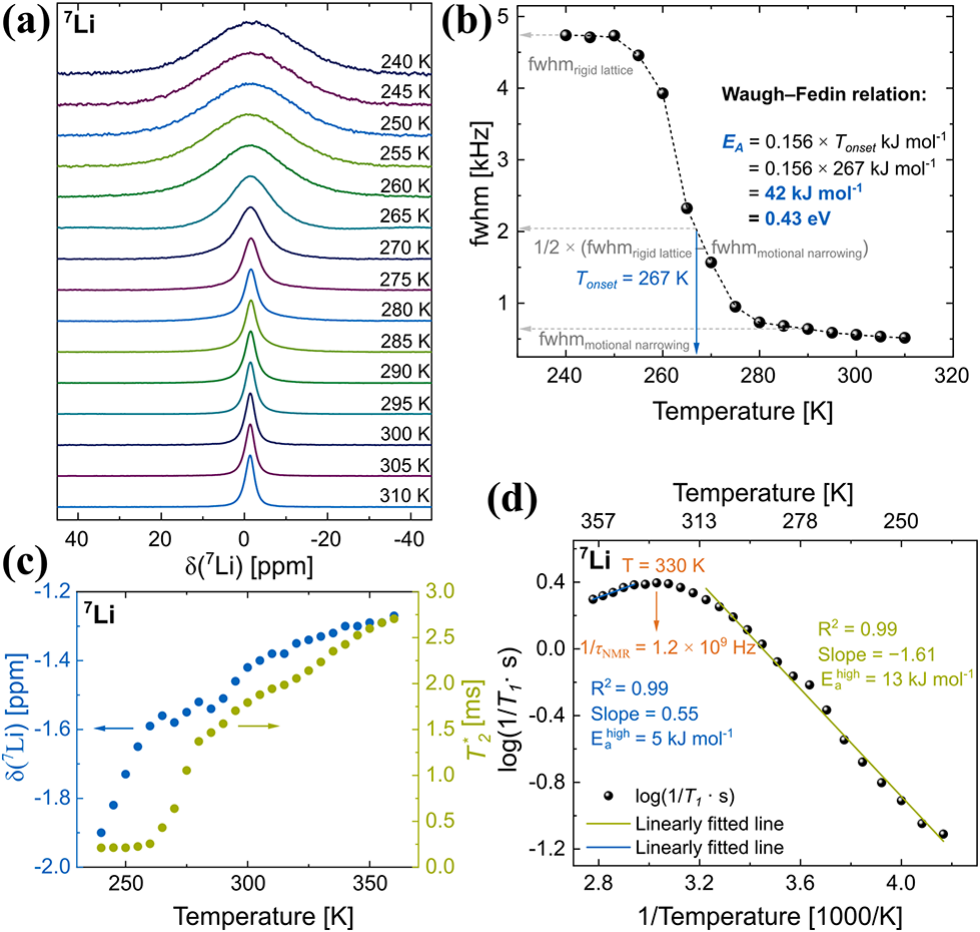
(a) Variable-temperature ^7^Li NMR nonspinning spectra of the dry membrane recorded between 240 and 310 K. (b) Temperature-dependent full width at half-maximum (fwhm) of the ^7^Li NMR resonance, used in Waugh-Fedin analysis to estimate an approximate activation energy (*E*_A_) of 42 kJ mol^– 1^ for Li-ion motion. (c) Temperature dependence of the ^7^Li isotropic chemical shift (δ) and effective spin–spin relaxation time (*T*_2_*). (d) Arrhenius-like plot of the diffusion-induced ^7^Li spin–lattice relaxation rates (1/*T*_1_) with a rate maximum near 330 K. Activation energies for the low- and high-temperature regimes were determined to be 13 and 5 kJ mol^– 1^, respectively.

Furthermore, this observed line width narrowing can also be attributed to an increase in the effective spin–spin relaxation time $\left(T_{2}^{*}\right)$ as shown in Figure 8c. A closer inspection also reveals a slightly higher frequency shift in the isotropic ^7^Li chemical shift, moving from −1.9 ppm to −1.3 ppm as the temperature increases from 240 to 360 K (Figure 8c).

To assess precise local activation energies and gain an understanding of Li-ion dynamics across different thermally activated regimes, variable-temperature (240 to 360 K)^7^Li spin–lattice relaxation (*T*_1_) measurements were performed. The spin–lattice relaxation rates (SLR, i.e., 1/*T*_1_) were analyzed using an Arrhenius-type representation, where ln­(1/*T*_1_) is plotted against 1000/*T* (*T* in K), as shown in Figure 8d. A characteristic diffusion-induced relaxation peak was observed with a maximum at ∼330 K, corresponding to the temperature where the mean jump rate of Li-ions (1/τ_NMR_) approaches the angular Larmor frequency (ω_0_), satisfying the condition ω_0_.τ_NMR_ ≈ 1.^49^ The estimated 1/τ_NMR_ at this temperature is 1.2 × 10^9^ Hz, placing it in the GHz range, which reflects rapid Li-ion exchange and suggests high ionic conductivity or fast local ion hopping behavior. The low-temperature side of the relaxation peak (ω_0_.τ_NMR_ ≫ 1) is sensitive to localized ion hopping processes.^50^ From the slope of this region, an activation energy $E_{a}^{low}$ of 13 kJ mol^–1^ was extracted, which is typically influenced by correlation effects such as structural disorder or Coulombic interactions. In contrast, the high-temperature side of the peak (ω_0_.τ_NMR_ ≪ 1) yields a lower activation energy $E_{a}^{high}$ of 5 kJ mol^–1^, indicative of favorable long-range Li-ion conduction. This high-temperature regime is often governed by the dimensionality and connectivity of the diffusion pathways.^50^

## 4 Conclusion

In summary, we fabricated free-standing, flexible, nanofibrous polymer electrolytes via a scalable, one-step electrospinning process. This process ensured the low crystallinity of the membranes and the dissolution of the conducting salt, LiTFSI, in the PAN/PEO polymer blend, as confirmed by *Raman* spectroscopy and XRD. Using porosity calculations and PEIS, we investigated the effects of plasticizer content (SN and PC), the relative humidity during electrospinning, and moisture, and the evaporation time between electrospinning and drying on the porosity and ionic conductivity. The optimal ratio of polymer to plasticizer to conducting salt was found to be 18:9:1. Electrospinning this membrane at a relative humidity between 19 and 33% yields a porosity between 33 and 44% and an optimal ionic conductivity of 0.1 mS cm^–1^ at 328 K and 0.028 mS cm^–1^ at room temperature. However, extremely low relative humidity during electrospinning leads to fiber fusion and a drop in porosity and conductivity.

Moreover, increasing the operation pressure from 0.6 to 2.1 MPa enhances the ionic conductivity further by half an order of magnitude. Nevertheless, increasing the operation pressure to 12.7 MPa does not significantly increase conductivity. However, increasing the pressure before the measurement to 62.5 MPa causes the fiber structure to collapse, resulting in a drop in conductivity.

Additionally, blending PAN into PEO enhances thermal stability and electrochemical stability. Despite PEO’s lower melting point, the membrane retains its fiber structure at 100 °C and exhibits an ionic conductivity of 2.3 × 10^–4^ S cm^–1^ at 95 °C. This was confirmed using SEM, TGA, and DSC measurements. The membranes exhibit an electrochemical stability window between 0 and 4.5 V, a critical current density of 0.5 mA cm^–2^, and a lithium transference number of 0.18. The lithium galvanostatic plating/stripping stability at 0.1 mA cm^–2^ is 120 cycles with the ability to recover from dendrites. XPS showed a decomposition after cycling of LiTFSI to LiF and other species as well as decomposition of the polymers.

Solid-state NMR studies revealed the sensitivity of the samples to moisture exposure and further highlighted the potential of these membranes for lithium-ion transport. This is supported by the observation of generally low activation energies for local Li-ion dynamics, which may be attributed to efficient long-range Li-ion conduction.

This work provides a one-step production method for a solid polymer electrolyte membrane that is safe, as well as thermally, mechanically, and electrochemically stable, and that exhibits sufficient ionic conductivity even at low operating pressures without further pretreatment.

## Supplementary Material



## Notes

Table of ionic conductivities and corresponding correlation plot, liquid NMR spectra and corresponding calculated molar starting material ratios, Arrhenius plots of Figures 3b and 5c, solid state ^1^H MAS, ^13^C­{^1^H} CP MAS and ^7^Li MAS NMR spectra, SEM images of a dry room and a precompressed membrane, long-term PEIS measurement, ionic conductivity of precompressed membrane, DSC curves for reference, chronoamperometry and PEIS for determining the lithium transference number, as well as XPS survey spectra (PDF)

## Abbreviations

ASSB: all-solid-state-battery
CP: cross-polarization
CV: cyclic voltammetry
DMF: dimethylformamide
DMSO: dimethyl sulfoxide
DSC: differential scanning calorimetry
ES: Electrospinning
fwhm: full width at half-maximum
LiTFSI: lithium bis­(trifluoromethanesulfonyl)­imide
MAS: magic angle spinning
MeCN: acetonitrile
NMR: nuclear magnetic resonance
OCV: open circuit voltage
PAN: polyacrylonitrile
PC: propylene carbonate
PEIS: potentiostatic electrochemical impedance spectroscopy
PEO: poly­(ethylene oxide); ppm, parts per million
R.H.: relative humidity
SLR: spin–lattice relaxation rates
SN: Succinonitrile
SPE: solid polymer electrolyte
SS: stainless steel; ssNMR, solid state NMR
TGA: thermogravimetric analysis
XPS: X-ray photoelectron spectroscopy.

## References

[1] Aziz S. B., Woo T. J., Kadir M., Ahmed H. M. (2018). A conceptual review
on polymer electrolytes and ion transport models. J. Sci: Adv. Mater. Dev..

[2] Arya A., Sharma A. L. (2020). A glimpse on all-solid-state
Li-ion battery (ASSLIB)
performance based on novel solid polymer electrolytes: a topical review. J. Mater. Sci..

[3] Jamal H., Khan F., Kim J. H., Kim E., Lee S. U., Kim J. H. (2024). Compact Solid Electrolyte Interface
Realization Employing
Surface-Modified Fillers for Long-Lasting, High-Performance All-Solid-State
Li-Metal Batteries. Small.

[4] Su X., Xu X.-P., Ji Z.-Q., Wu J., Ma F., Fan L.-Z. (2024). Polyethylene Oxide-Based Composite
Solid Electrolytes
for Lithium Batteries: Current Progress, Low-Temperature and High-Voltage
Limitations, and Prospects. Electrochem. Energy
Rev..

[5] Kirchberger A. M., Walke P., Venturini J., van Wüllen L., Nilges T. (2025). Highly Conductive PEO/PAN-Based SN-Containing
Electrospun
Membranes as Solid Polymer Electrolytes. Membranes.

[6] Li X., Deng Y., Li K., Yang Z., Hu X., Liu Y., Zhang Z. (2023). Advancements
in Performance Optimization of Electrospun
Polyethylene Oxide-Based Solid-State Electrolytes for Lithium-Ion
Batteries. Polymers.

[7] Choi B. K., Kim Y. W., Shin H. K. (2000). Ionic conduction
in PEO–PAN
blend polymer electrolytes. Electrochim. Acta.

[8] Liang Y.-H., Wang C.-C., Chen C.-Y. (2007). Conductivity and
characterization
of plasticized polymer electrolyte based on (polyacrylonitrile-b-polyethylene
glycol) copolymer. J. Power Sources.

[9] Yuan F., Chen H.-Z., Yang H.-Y., Li H.-Y., Wang M. (2005). PAN–PEO
solid polymer electrolytes with high ionic conductivity. Mater. Chem. Phys..

[10] Xue J., Wu T., Dai Y., Xia Y. (2019). Electrospinning and Electrospun Nanofibers:
Methods, Materials, and Applications. Chem.
Rev..

[11] Freitag K. M., Kirchhain H., van Wüllen L., Nilges T. (2017). Enhancement of Li Ion
Conductivity by Electrospun Polymer Fibers and Direct Fabrication
of Solvent-Free Separator Membranes for Li Ion Batteries. Inorg. Chem..

[12] Walke P., Freitag K. M., Kirchhain H., Kaiser M., van Wüllen L., Nilges T. (2018). Electrospun Li­(TFSI)@Polyethylene
Oxide Membranes as
Solid Electrolytes. Z. Anorg. Allg. Chem..

[13] Polu A. R., Kim K., Kareem A. A., Kim D., Song S., Savilov S. V., Singh P. K. (2025). Impact of tetracyanoethylene plasticizer on PEO based
solid polymer electrolytes for improved ionic conductivity and solid-state
lithium-ion battery performance. J. Power Sources.

[14] Zhang L., Hsieh Y.-L. (2006). Nanoporous ultrahigh
specific surface polyacrylonitrile
fibres. Nanotechnology.

[15] Kim C. S., Oh S. M. (2000). Importance of donor
number in determining solvating ability of polymers
and transport properties in gel-type polymer electrolytes. Electrochim. Acta.

[16] Abdollahi S., Ehsani M., Morshedian J., Khonakdar H. A., Reuter U. (2018). Structural and electrochemical properties
of PEO/PAN
nanofibrous blends: Prediction of graphene localization. Polym. Compos..

[17] Ma Y., Wan J., Yang Y., Ye Y., Xiao X., Boyle D. T., Burke W., Huang Z., Chen H., Cui Y. (2022). Scalable, Ultrathin,
and High-Temperature-Resistant Solid Polymer
Electrolytes for Energy-Dense Lithium Metal Batteries. Adv. Energy Mater..

[18] Zhang Y., Wang H., Yang Y., Xie J., Deng Q., Zou W., Zhou A., Li J. (2023). Polyacrylonitrile fibers network
reinforced polymer electrolyte with Li-Sn alloy layer protected Li
anode toward ultra-long cycle lifespan for room-temperature solid-state
batteries. Chem. Eng. J..

[19] Gao L., Li J., Sarmad B., Cheng B., Kang W., Deng N. (2020). A 3D polyacrylonitrile
nanofiber and flexible polydimethylsiloxane macromolecule combined
all-solid-state composite electrolyte for efficient lithium metal
batteries. Nanoscale.

[20] He F., Tang W., Zhang X., Deng L., Luo J. (2021). High Energy
Density Solid State Lithium Metal Batteries Enabled by Sub-5 μm
Solid Polymer Electrolytes. Adv. Mater..

[21] Zhang Z., YingHuang, Zhang G., Chao L. (2021). Three–dimensional fiber network reinforced polymer electrolyte
for dendrite–free all–solid–state lithium metal
batteries. Energy Storage Mater..

[22] Li D., Chen L., Wang T., Fan L.-Z. (2018). 3D Fiber-Network-Reinforced
Bicontinuous Composite Solid Electrolyte for Dendrite-free Lithium
Metal Batteries. ACS Appl. Mater. Interfaces.

[23] Bloch F. (1946). Nuclear Induction. Phys. Rev..

[24] Pines A., Gibby M. G., Waugh J. S. (1972). Proton-Enhanced
Nuclear Induction
Spectroscopy. A Method for High Resolution NMR of Dilute Spins in
Solids. J. Chem. Phys..

[25] Hartmann S. R., Hahn E. L. (1962). Nuclear Double Resonance
in the Rotating Frame. Phys. Rev..

[26] Bernard G. M., Goyal A., Miskolzie M., McKay R., Wu Q., Wasylishen R. E., Michaelis V. K. (2017). Methylammonium lead chloride: A sensitive
sample for an accurate NMR thermometer. J. Magn.
Reson..

[27] Scheers J., Niedzicki L., Zukowska G. Z., Johansson P., Wieczorek W., Jacobsson P. (2011). Ion-ion and ion-solvent interactions
in lithium imidazolide electrolytes studied by Raman spectroscopy
and DFT models. Phys. Chem. Chem. Phys..

[28] Brodin A., Jacobsson P. (2009). On the structure
of archetypal polymer electrolyte
PEO: LiCF_3_SO_3_. Ukr. J.
Phys..

[29] Hiraoka K., Seki S. (2023). Operando Raman Spectroscopy
for Evaluating Concentration Changes
in Li- and Na-Based Solid Polymer Electrolytes. J. Phys. Chem. C.

[30] Spranger R. J., van Wüllen L., Kirchberger A. M., Nilges T. (2025). Highly-conductive mixed
PEO/PAN-based membranes for solid state Li-ion batteries via electro-spinning
and hot-press synthesis routes. Z. Anorg. Allg.
Chem..

[31] Zhao Y., Wang L., Zhou Y., Liang Z., Tavajohi N., Li B., Li T. (2021). Solid Polymer
Electrolytes with High Conductivity and
Transference Number of Li Ions for Li-Based Rechargeable Batteries. Adv. Sci..

[32] Espejo J., Zellmann-Parrotta C. O., Sarkar D., Che A., Michaelis V. K., Williams V. E., Ling C.-C. (2024). Unprecedented Cubic Mesomorphic Behaviour
of Crown-Ether Functionalized Amphiphilic Cyclodextrins. Chem.–Eur. J..

[33] Di
Muzio S., Paolone A., Brutti S. (2021). Thermodynamics of the
Hydrolysis of Lithium Salts: Pathways to the Precipitation of Inorganic
SEI Components in Li-Ion Batteries. J. Electrochem.
Soc..

[34] Meyer B. M., Leifer N., Sakamoto S., Greenbaum S. G., Grey C. P. (2005). High Field Multinuclear NMR Investigation of the SEI
Layer in Lithium Rechargeable Batteries. Electrochem.
Solid-State Lett..

[35] Zhang J., Fu J., Lu P., Hu G., Xia S., Zhang S., Wang Z., Zhou Z., Yan W., Xia W., Wang C., Sun X. (2025). Challenges and Strategies of Low-Pressure
All-Solid-State Batteries. Adv. Mater..

[36] Zhang Z., Lv M., Huang D., Jia P., Sun D., Li W. (2013). Isobaric Vapor–Liquid
Equilibrium for the Extractive Distillation of Acetonitrile + Water
Mixtures Using Dimethyl Sulfoxide at 101.3 kPa. J. Chem. Eng. Data.

[37] Wu X., Zhao G., Wang X., Liu W. (2017). Preparation of High-Temperature
Lubricants by Blending Castor Oil with Lithium Bis­(trifluoromethylsulfonyl)­imide. Tribol. Lett..

[38] Salles V., Bernard S., Brioude A., Cornu D., Miele P. (2010). A new class
of boron nitride fibers with tunable properties by combining an electrospinning
process and the polymer-derived ceramics route. Nanoscale.

[39] Bruce P. G., Evans J., Vincent, Colin A. (1988). Conductivity and transference number
measurements on polymer electrolytes. Solid
State Ionics.

[40] Guo Z., Liao S., Li F., Shi L., Su B., Mu J., Wang Y., Huang Z., Xu F., Guan C. (2024). Constructing
efficient lithium salt dissociation solid-state electrolytes with
high conductivity and transference number of Li ions for stable all-solid-state
lithium batteries. Chem. Eng. J..

[41] Kim E., Jamal H., Jeon I., Khan F., Chun S.-E., Kim J. H. (2023). Functionality of
1-Butyl-2,3-Dimethylimidazolium Bromide
(BMI-Br) as a Solid Plasticizer in PEO-Based Polymer Electrolyte for
Highly Reliable Lithium Metal Batteries. Adv.
Energy Mater..

[42] Lu Y., Zhao C.-Z., Yuan H., Cheng X.-B., Huang J.-Q., Zhang Q. (2021). Critical Current
Density in Solid-State Lithium Metal Batteries:
Mechanism, Influences, and Strategies. Adv.
Funct. Mater..

[43] Huang X., Wu L., Huang Zhen, Lin Jiu, Xu Xiaoxiong (2020). Characterization
and testing of key electrical and electrochemical properties of lithium-ion
solid electrolytes. Energy Storage Sci. Technol..

[44] Xu C., Sun B., Gustafsson T., Edström K., Brandell D., Hahlin M. (2014). Interface
layer formation in solid polymer electrolyte lithium batteries: an
XPS study. J. Mater. Chem. A.

[45] Seidl L., Grissa R., Zhang L., Trabesinger S., Battaglia C. (2022). Unraveling the Voltage-Dependent
Oxidation Mechanisms
of Poly­(Ethylene Oxide)-Based Solid Electrolytes for Solid-State Batteries. Adv. Mater. Interfaces.

[46] Meng H., Xu T., Gao M., Bai J., Li C. (2021). An oil-contamination-resistant
PVP / PAN electrospinning membrane for high-efficient oil–water
mixture and emulsion separation. J. Appl. Polym.
Sci..

[47] Lacey M. J., Yalamanchili A., Maibach J., Tengstedt C., Edström K., Brandell D. (2016). The Li–S battery: an investigation
of redox shuttle and self-discharge behaviour with LiNO 3 -containing
electrolytes. RSC Adv..

[48] Waugh J. S., Fedin E. I. (1963). Determination of
hindered-rotation barriers in solids. Sov. Phys.
Solid State.

[49] Wilkening M., Heitjans P. (2012). From micro
to macro: access to long-range Li+ diffusion
parameters in solids via microscopic (6, 7) Li spin-alignment echo
NMR spectroscopy. ChemPhyschem.

[50] Sarkar D., Bhattacharya A., Meyer J., Kirchberger A. M., Mishra V., Nilges T., Michaelis V. K. (2023). Unraveling
Sodium-Ion Dynamics in Honeycomb-Layered Na_2_Mg_x_Zn_2-x_TeO_6_ Solid Electrolytes with Solid-State
NMR. J. Am. Chem. Soc..

