# Supplementary material for: Performance Optimization of Electrospun Lithium-Ion Conducting PAN/PEO Solid Polymer Electrolyte
Source: Inorg Chem. 2025 Sep 25;64(39):19752–63. doi: 10.1021/acs.inorgchem.5c03238 (PMC12505261; doi:10.1021/acs.inorgchem.5c03238)
Supplement: Supplementary file 1 [file ic5c03238_si_001.pdf]

Supporting Information

Performance Optimization of Electrospun Lithium-Ion Conducting PAN/PEO Solid Polymer Electrolyte

Elisabeth B. Springl[1,2], Diganta Sarkar[3], Marvin Mühlau[1,2], Vladimir K. Michaelis[3], Tom Nilges[,1]*

[1] School of Natural Sciences (NAT), Department of Chemistry, Technische Universität München, Lichtenbergstraße 4, 85748 Garching bei München, Germany

[2] TUMint.Energy Research GmbH, Lichtenbergstraße 4, 85748 Garching bei München, Germany

[3] Department of Chemistry, University of Alberta, Edmonton, Alberta T6G 2G2, Canada

[*] Corresponding author: tom.nilges@tum.de

Table S1: Ionic conductivities at room temperature in relation to their relative humidity during electrospinning, calculated porosity, and activation energy, as determined by the Arrhenius equation. *was densified artificially by rolling. ‘was analyzed with ssNMR.

**Conductivity at 25 °C [S cm^{-1}]**	**Relative humidity [%]**	**Calculated porosity [%]**	**Activation energy [eV]**
2.80×10^{-5}	30	44.4	0.40
9.76×10^{-6}	22	33.9	0.51’
8.87×10^{-6}	19	38.0	0.54
6.08×10^{-6}	21	33.8	0.54
5.05×10^{-6}	33	44.5	0.49
1.69×10^{-6}	45	36.7*	0.64
1.81×10^{-6}	42	57.7	0.58
1.86×10^{-6}	38	61.6	0.57
1.16×10^{-6}	41	62.2	0.61
9.92×10^{-7}	37	56.7	0.63
2.15×10^{-7}	0	12.7	0.35’

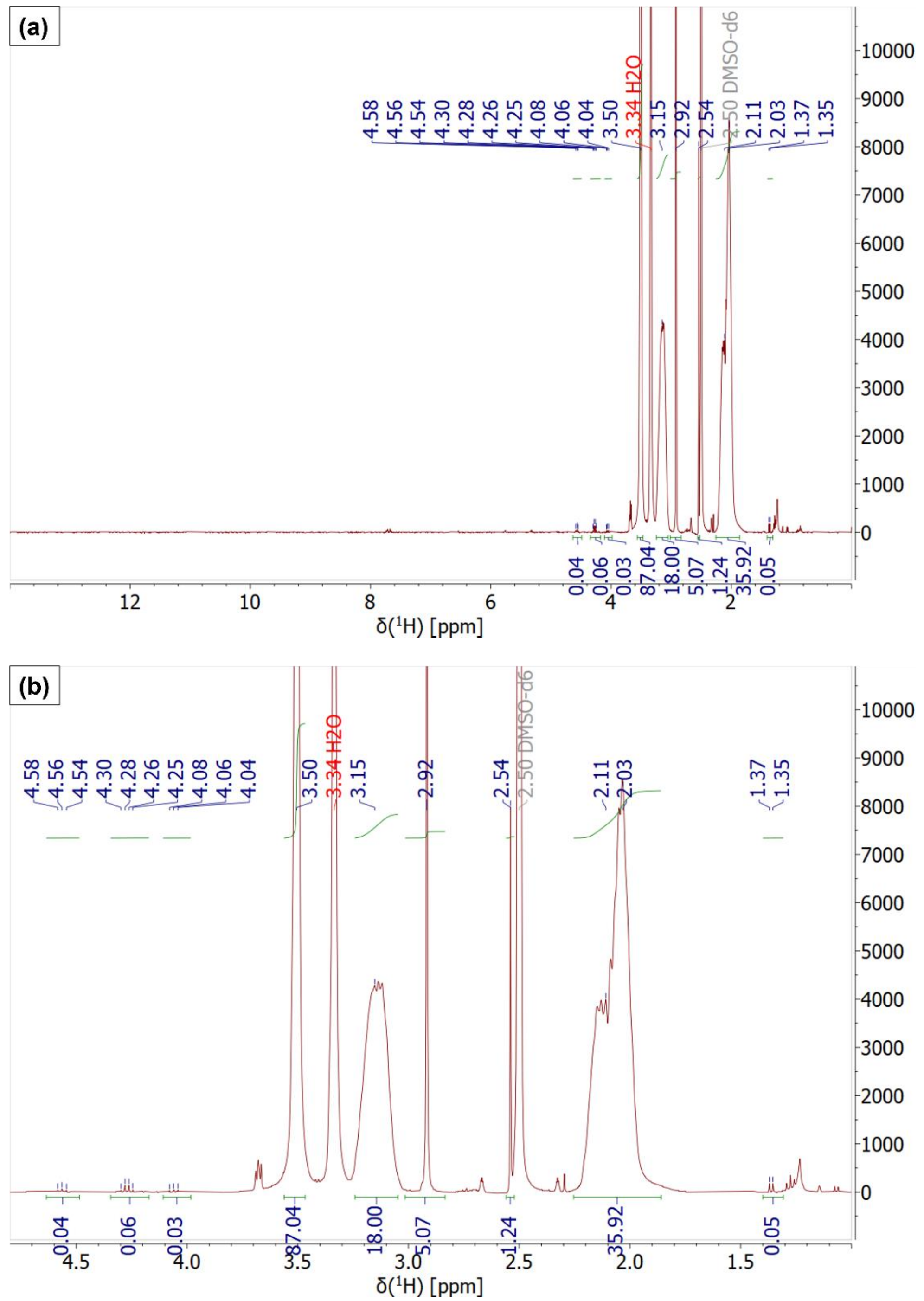

Figure S1: ^{1}H NMR spectrum of an 18:9:1 SPE (a) full spectrum and (b) zoom. Water residues at 3.34 ppm belong to the not dried, deuterated solvent.

Table S2: Weighed in masses and molar ratio of the starting materials of an 18:9:1 SPE as well as their molar and mass ratios determined by liquid ^{1}H NMR in d^{6}-DMSO (see Figure S1). * As LiTFSI can't be analyzed by ^{1}H NMR and is expected to remain stable during electrospinning and drying, no weight loss of LiTFSI was assumed.

	PAN	PEO	PC	SN	LiTFSI
Weighed in mass	0.2500 g	0.2500 g	0.2405 g	0.2274 g	0.1657 g
Weighed in molar ratio	18	21.7	9	10.8	2
Molar ratio from ^{1}H NMR	18.00	21.76	0.035	1.27	- *
Weight ratio from NMR	36.02%	36.12%	0.13%	3.85%	23.88%

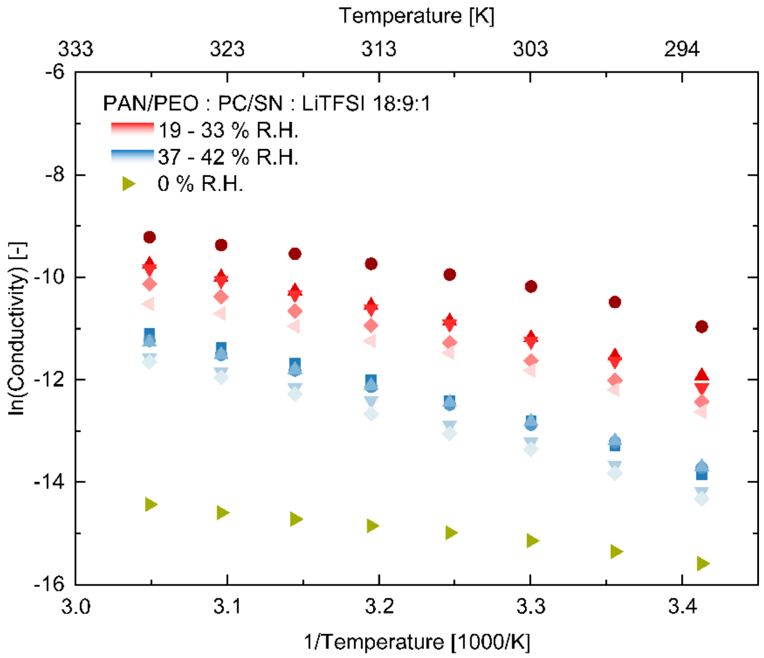

Figure S2: Arrhenius plot of SPEs depending on the relative humidity (R.H.) during electrospinning. Activation energies are given in Table S1.

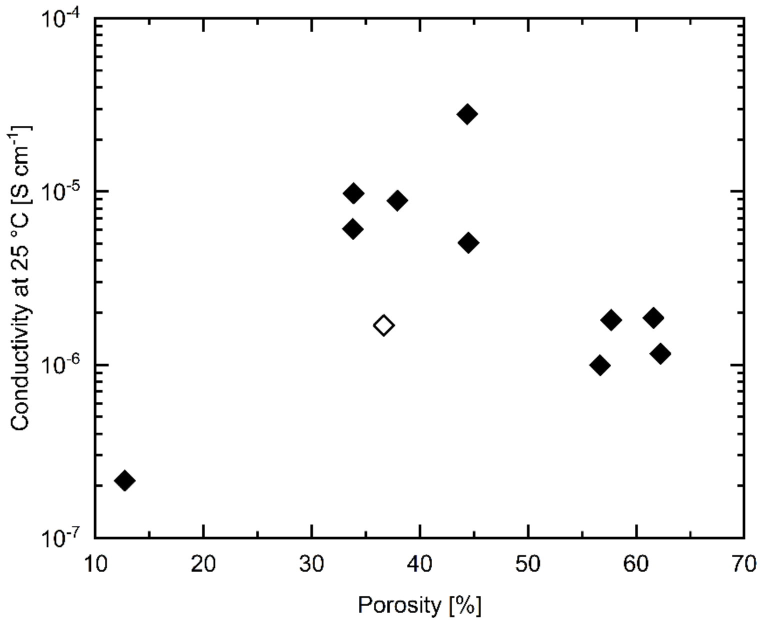

Figure S3: Correlation between calculated porosity and room temperature ionic conductivity for normally dried membranes (filled diamonds) and a pressed membrane before drying (empty diamonds).

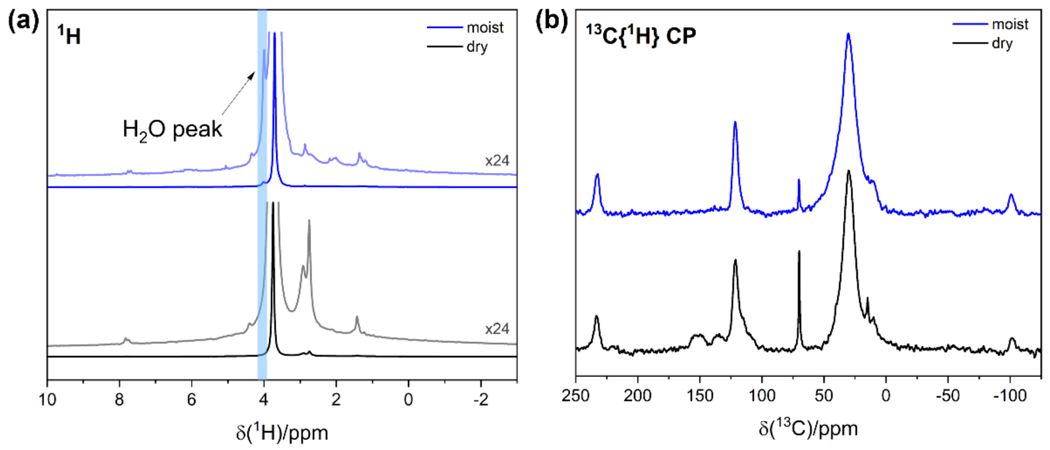

Figure S4: Solid-state (a) ^{1}H MAS and (b) ^{13}C{^{1}H} CP MAS NMR spectra (acquired at 11.75 T using an MAS frequency of 14 kHz) of an SPE spun in the dry room at 0 % R.H. before (dry) and after moisture exposure on air (moist). ^{1}H NMR signal from water is highlighted in blue.

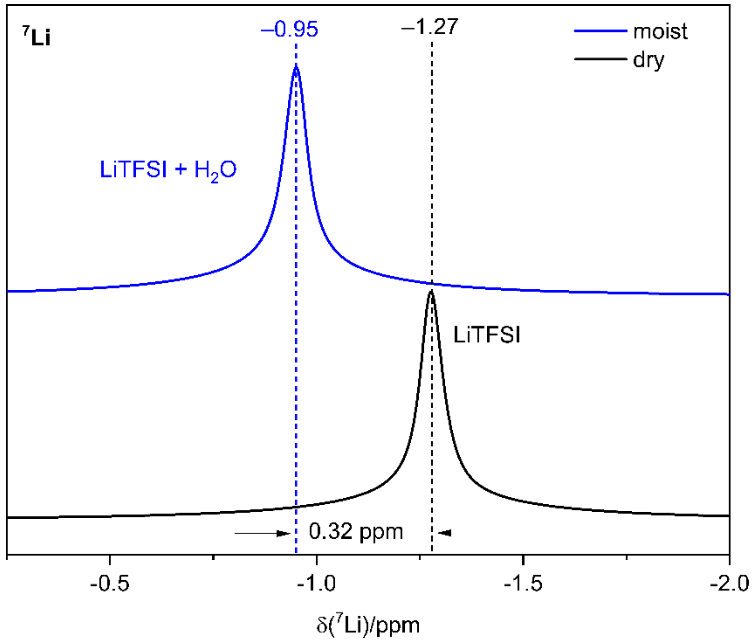

Figure S5: Solid-state ^{7}Li MAS NMR (at 11.75 T with an MAS frequency = 14 kHz) of an 18:9:1 SPE spun in the dry room at 0 % R.H. before (dry) and after moisture exposure on air (moist); the dotted lines act as a guide to the eye.

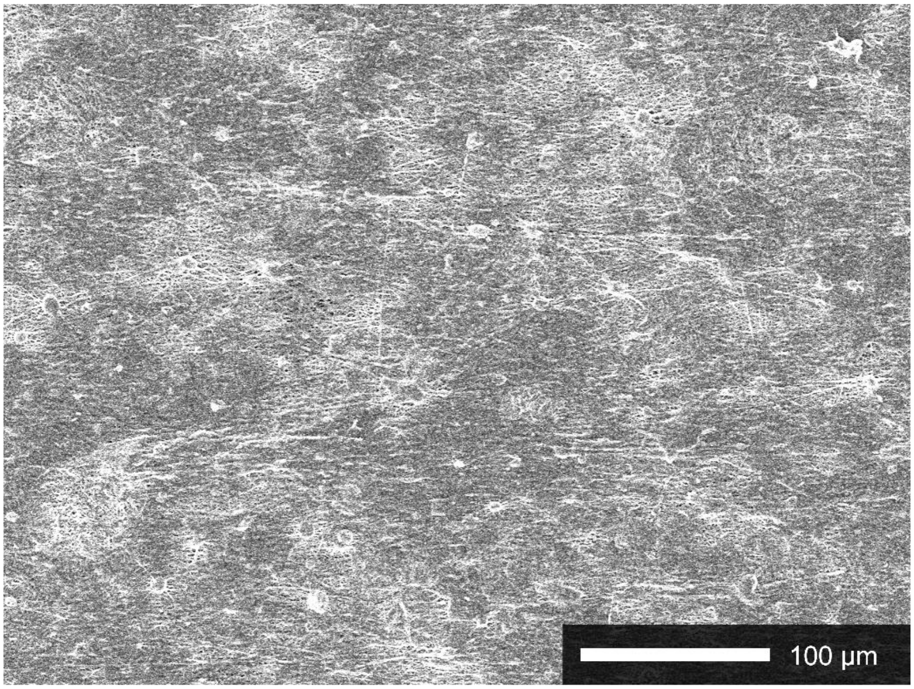

Figure S6: SEM image of an 18:9:1 SPE electrospun in the dry room.

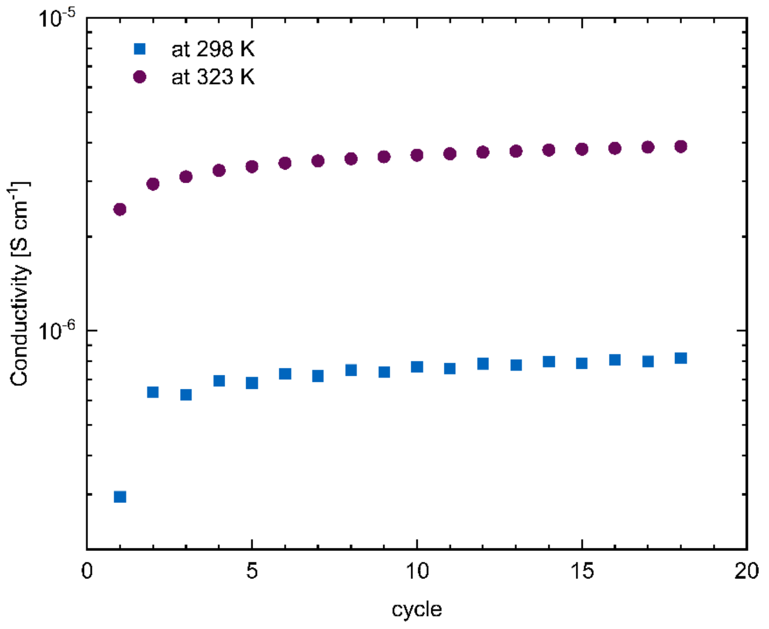

Figure S7: Long-term PEIS measurement over 18 heating (odd cycle numbers) and cooling (even cycle numbers) cycles at 298 K and 323 K. The ionic conductivity seems to increase in the beginning because the membrane is compressed during the first heating cycle. The thickness used for the conductivity calculation is measured after the last cycle.

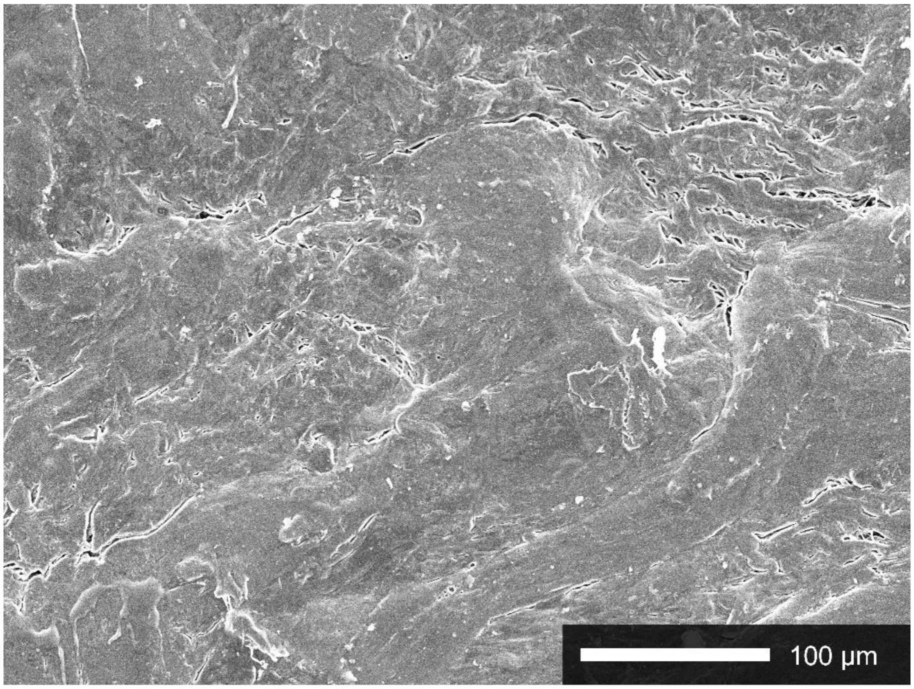

Figure S8: SEM image of an electrospun 18:9:1 SPE, which was precompressed with 62.5 MPa.

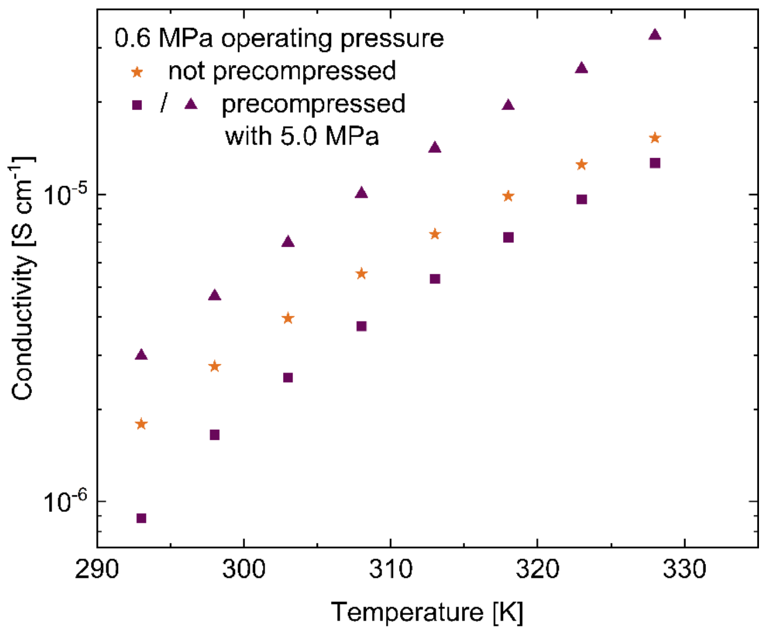

Figure S9: Temperature-dependent ionic conductivity of different spots of an 18:9:1 SPE membrane either not precompressed (orange) or precompressed at 5.0 MPa (purple). All membrane discs are measured at an operating pressure of 0.6 MPa. As the ionic conductivity of the precompressed membrane discs varies by half an order of magnitude, one must be careful comparing different membrane parts with one another.

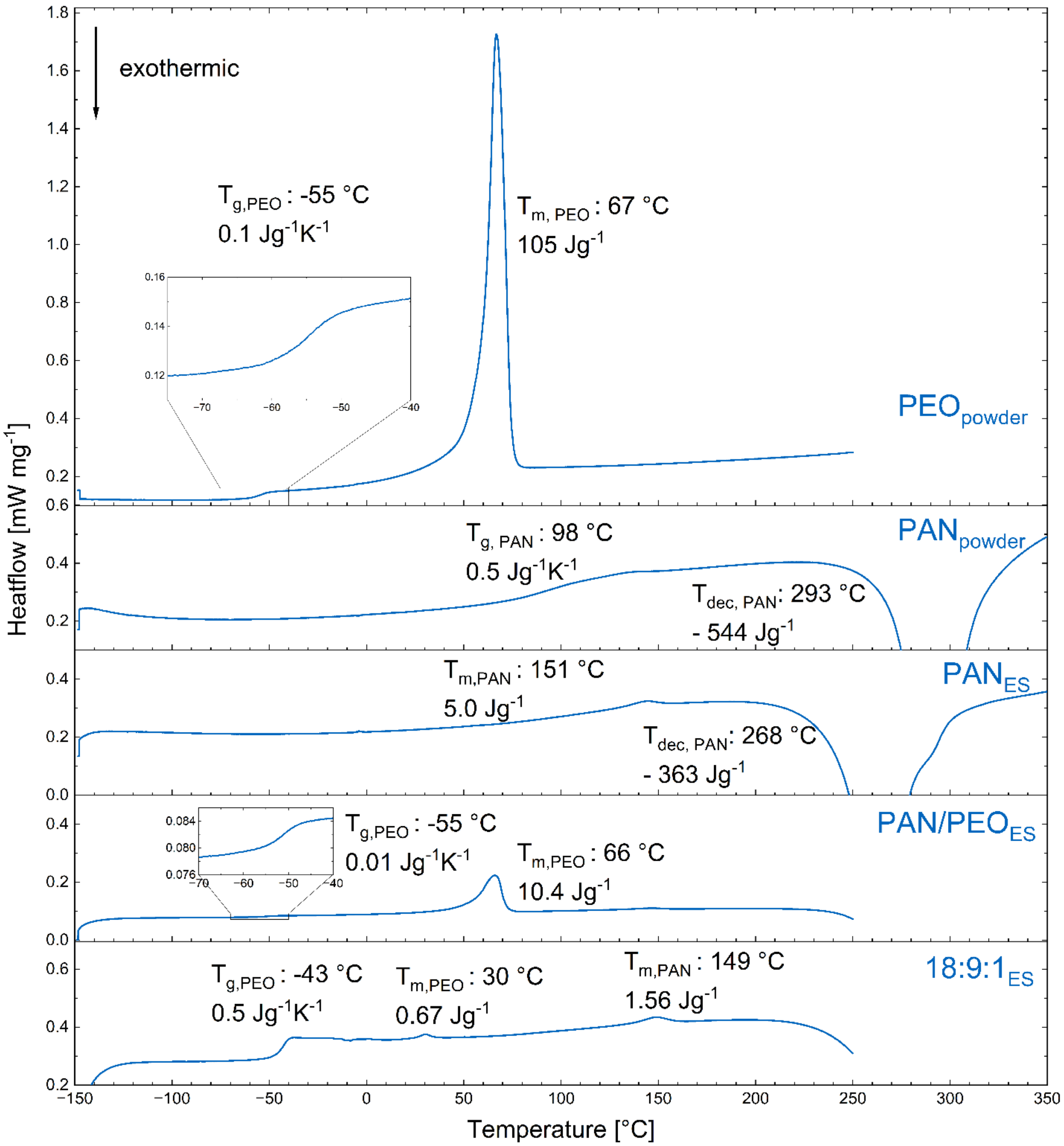

Figure S10: DSC curves of PEO powder, PAN powder, electrospun PAN, electrospun PAN/PEO without plasticizer, and LiTFSI, as well as of an electrospun SPE membrane with the composition 18:9:1. Additionally, their respective melting, glass-transition and decomposition temperatures with corresponding integrals.

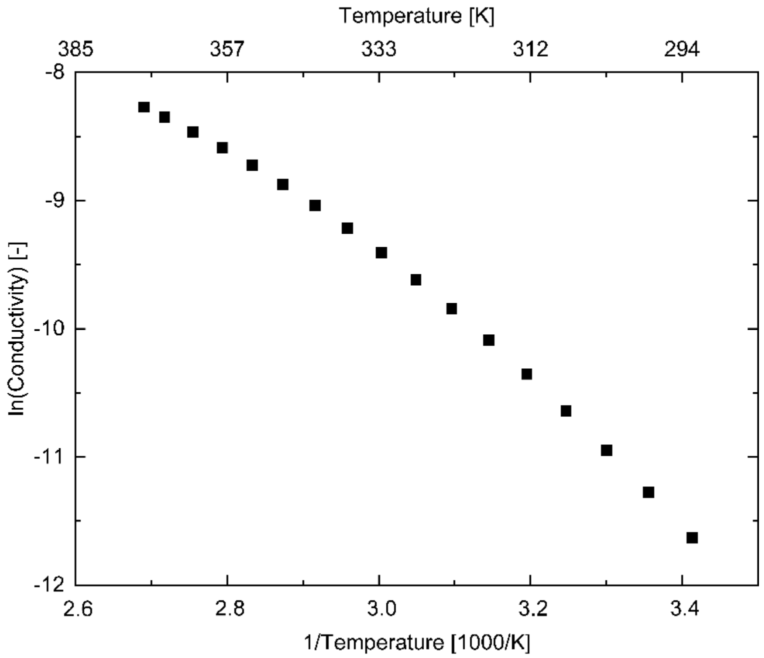

Figure S11: Arrhenius plot of an SPE membrane heated up to 95 °C.

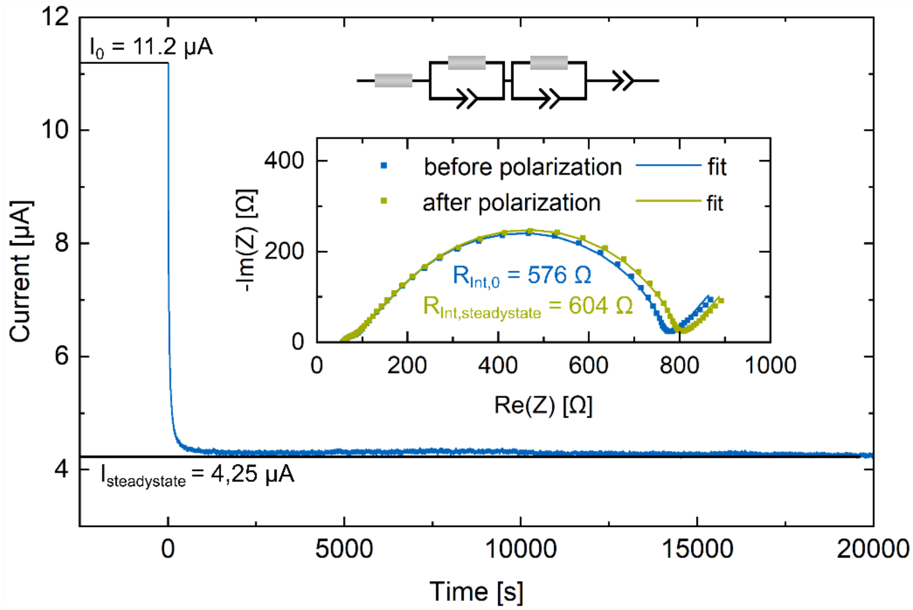

Figure S12: Chronoamperometry profile (ΔV = 10 mV) of symmetric Li|SPE|Li cells of membranes with a PAN/PEO : PC/SN : LiTFSI composition of 18:9:1, as well as impedance before and after polarization (inset) with corresponding equivalent circuit for fitting the impedance data.

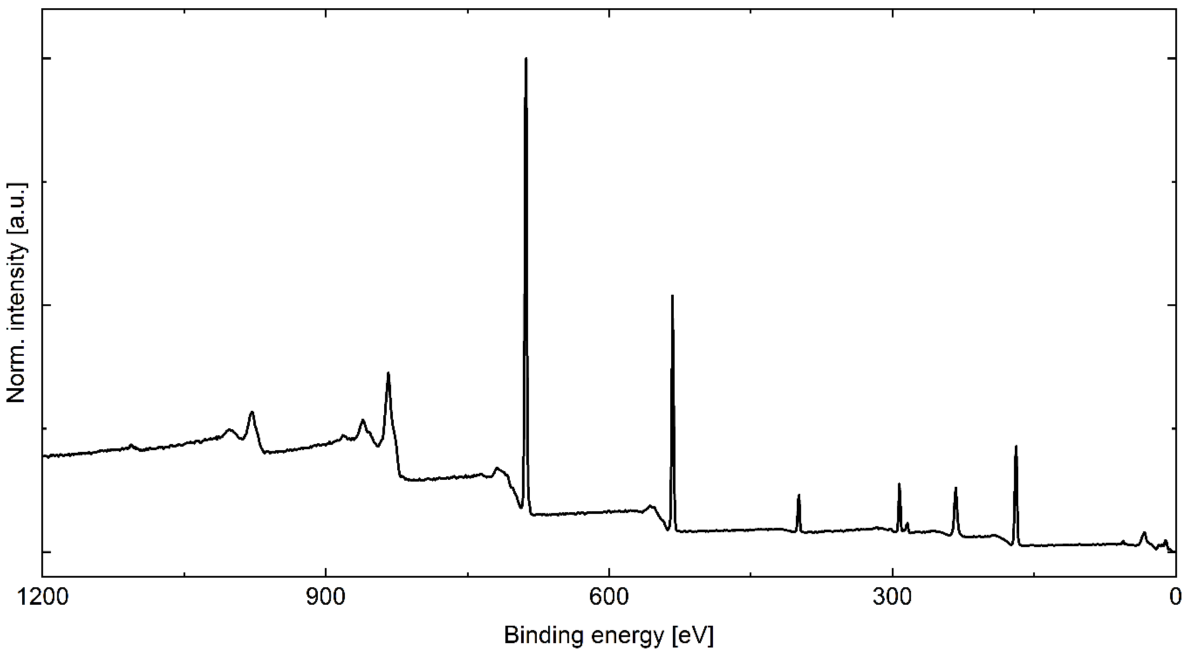

Figure S13: XPS survey spectrum of LiTFSI. The elements observed are fluorine, oxygen, nitrogen, carbon and sulfur, as discussed in Figure 7, and lithium, showing one sharp peak.

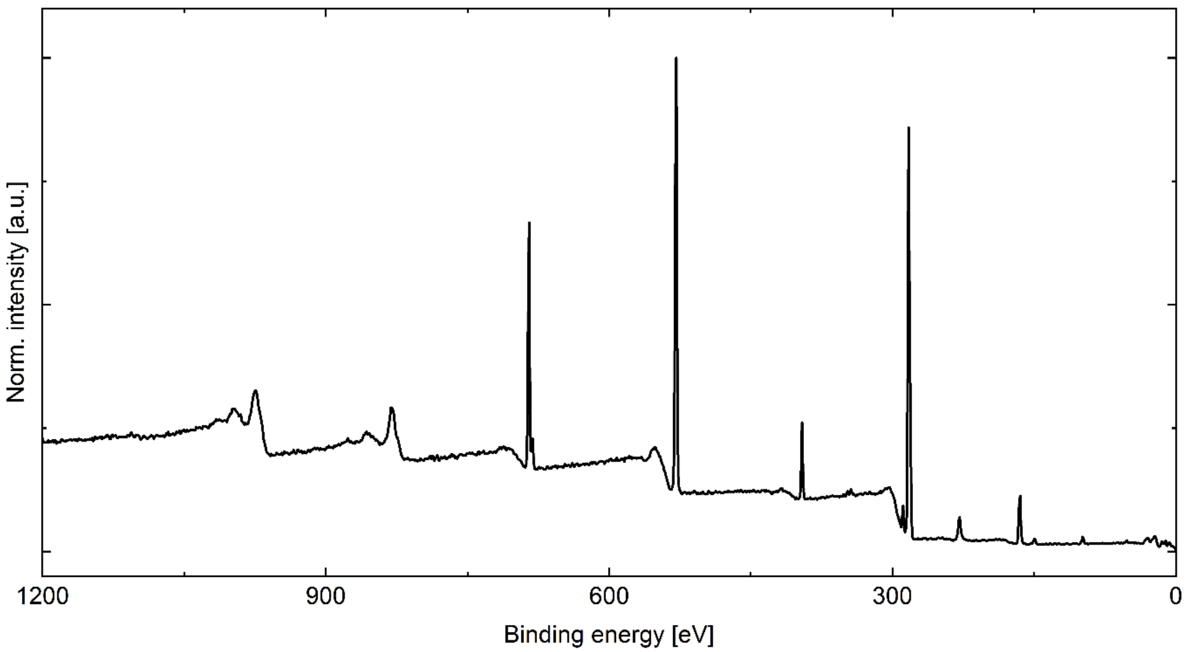

Figure S14: XPS survey spectrum of the noncycled SPE. The elements observed are fluorine, oxygen, nitrogen, carbon and sulfur, as discussed in Figure 7, and lithium, showing one sharp peak.

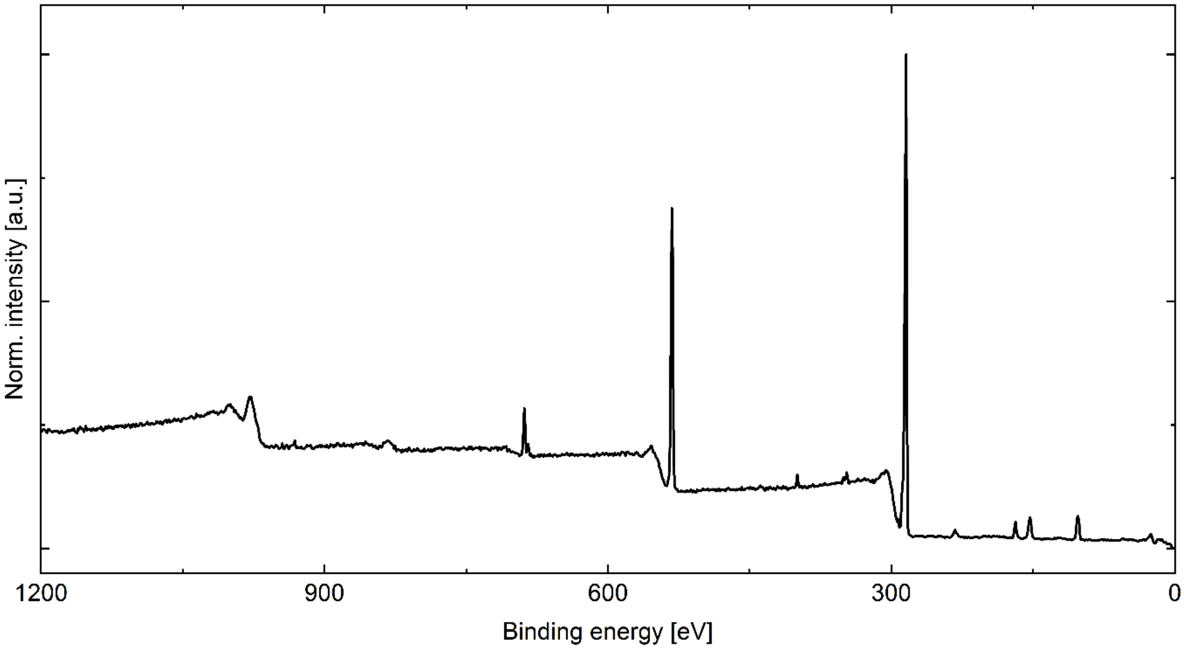

Figure S15: XPS survey spectrum of the cycled SPE. The elements observed are fluorine, oxygen, nitrogen, carbon and sulfur, as discussed in Figure 7, and lithium, showing one sharp peak.
